# Lymphopenia and risk of infection and infection-related death in 98,344 individuals from a prospective Danish population-based study

**DOI:** 10.1371/journal.pmed.1002685

**Published:** 2018-11-01

**Authors:** Marie Warny, Jens Helby, Børge Grønne Nordestgaard, Henrik Birgens, Stig Egil Bojesen

**Affiliations:** 1 Department of Hematology, Herlev and Gentofte Hospital, Copenhagen University Hospital, Herlev, Denmark; 2 Faculty of Health and Medical Science, University of Copenhagen, Copenhagen, Denmark; 3 Department of Clinical Biochemistry, Herlev and Gentofte Hospital, Copenhagen University Hospital, Herlev, Denmark; 4 Copenhagen General Population Study, Herlev and Gentofte Hospital, Copenhagen University Hospital, Herlev, Denmark; Heinrich Petter Institute, GERMANY

## Abstract

**Background:**

Neutropenia increases the risk of infection, but it is unknown if this also applies to lymphopenia. We therefore tested the hypotheses that lymphopenia is associated with increased risk of infection and infection-related death in the general population.

**Methods and findings:**

Of the invited 220,424 individuals, 99,191 attended examination. We analyzed 98,344 individuals from the Copenhagen General Population Study (Denmark), examined from November 25, 2003, to July 9, 2013, and with available blood lymphocyte count at date of examination. During a median of 6 years of follow-up, they developed 8,401 infections and experienced 1,045 infection-related deaths. Due to the completeness of the Danish civil and health registries, none of the 98,344 individuals were lost to follow-up, and those emigrating (*n =* 385) or dying (*n =* 5,636) had their follow-up truncated at the day of emigration or death. At date of examination, mean age was 58 years, and 44,181 (44.9%) were men. Individuals with lymphopenia (lymphocyte count < 1.1 × 10^9^/l, *n =* 2,352) compared to those with lymphocytes in the reference range (1.1–3.7 × 10^9^/l, *n =* 93,538) had multivariable-adjusted hazard ratios of 1.41 (95% CI 1.28–1.56) for any infection, 1.31 (1.14–1.52) for pneumonia, 1.44 (1.15–1.79) for skin infection, 1.26 (1.02–1.56) for urinary tract infection, 1.51 (1.21–1.89) for sepsis, 1.38 (1.01–1.88) for diarrheal disease, 2.15 (1.16–3.98) for endocarditis, and 2.26 (1.21–4.24) for other infections. The corresponding hazard ratio for infection-related death was 1.70 (95% CI 1.37–2.10). Analyses were adjusted for age, sex, smoking status, cumulative smoking, alcohol intake, body mass index, plasma C-reactive protein, blood neutrophil count, recent infection, Charlson comorbidity index, autoimmune diseases, medication use, and immunodeficiency/hematologic disease. The findings were robust in all stratified analyses and also when including only events later than 2 years after first examination. However, due to the observational design, the study cannot address questions of causality, and our analyses might theoretically have been affected by residual confounding and reverse causation. In principle, fluctuating lymphocyte counts over time might also have influenced analyses, but lymphocyte counts in 5,181 individuals measured 10 years after first examination showed a regression dilution ratio of 0.68.

**Conclusions:**

Lymphopenia was associated with increased risk of hospitalization with infection and increased risk of infection-related death in the general population. Notably, causality cannot be deduced from our data.

## Abstract 2

Stig Bojesen and colleagues highlight the increased risk of future infection or death for those with lymphopenia using data from a large Danish cohort.

## Author summary

**Why was this study done?:**

- Neutropenia and lymphopenia are low concentrations in the blood of the white blood cells—neutrophil granulocytes and lymphocytes, respectively; both are important for protecting against infections.
- Individuals with neutropenia have a well-documented increased risk of infection.
- It is currently unknown whether lymphopenia is associated with risk of infection in individuals from the general population.

**What did the researchers do and find?:**

- We investigated whether a low lymphocyte count could predict risk of later hospitalization due to an infection or risk of death due to an infection.
- The study population consisted of 98,344 individuals from the general population in Copenhagen, Denmark. All included individuals answered a questionnaire on lifestyle and health, had a physical examination, and had blood samples drawn at the date of examination.
- We found that lymphopenia in the general population was associated with a 1.4-fold increased risk of infection and a 1.7-fold increased risk of infection-related death.

**What do these findings mean?:**

- The study design cannot address questions of causality; however, risk of infection was increased in individuals with lymphopenia even 2 years after blood sampling, indicating that undiagnosed infection or comorbidity is not likely to be the only explanation for the results.
- Physicians are generally not recommended to intervene in patients with lymphopenia without an associated diagnosed disease. This might deserve reconsideration, since individuals with lymphopenia have increased risk of infection and infection-related death.

## Introduction

Neutropenia (neutrophil count < 0.5 × 10^9^/l) is associated with increased risk of infection [1,2], and the risk increases with lower and lower neutrophil counts [3,4]. In contrast, it is unknown whether lymphopenia also is associated with increased risk of infection in individuals from the general population. Importantly, physicians are generally not recommended to intervene in patients with lymphopenia without an associated diagnosed disease. Lymphopenia in the general population is typically discovered by chance when doing routine blood examination, and is often managed by the general practitioner. If asymptomatic, these patients are usually not referred for further examination, and isolated lymphopenia is generally not considered as threatening as neutropenia.

Lymphopenia may be caused by primary conditions such as congenital immunodeficiency disorders [5] or secondary causes such as malnutrition [6], alcohol abuse [7,8], medications [9], malignancies [10–12], systemic autoimmune diseases [13–15], and infections [13,16,17]. Diseases that cause lymphopenia are typically associated with an increased predisposition to various infections, either directly as a result of the lymphopenia-associated immune suppression or because of the underlying disease.

In this study, which included 98,344 individuals from the general population, we tested the hypotheses that lymphopenia is associated with increased risk of infection and infection-related death.

## Methods

### Participants

**Fig 1:**
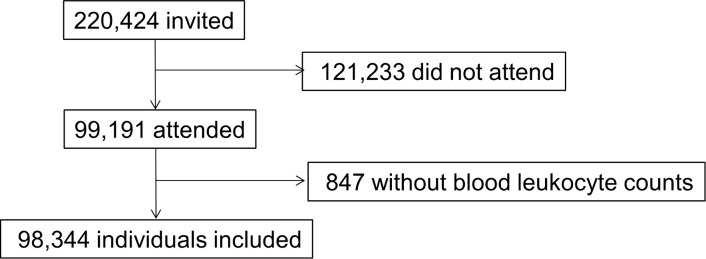
Flow chart of individuals from the Copenhagen General Population Study.

We studied 98,344 individuals from the prospective Copenhagen General Population Study [18–20], recruited from November 25, 2003, to July 9, 2013 (Fig 1). Individuals aged 20–100 years were invited from the general population, using the Danish Civil Registration System [21]. At the date of examination, all individuals completed a questionnaire on lifestyle and health (S1 and S2 Texts), had a physical examination, and provided blood samples. All individuals were white and of Danish descent. Due to the completeness of the Danish civil and health registries, no individuals were lost to follow-up, and those emigrating (*n =* 385) or dying (*n =* 5,636) had their follow-up truncated at the day of emigration or death. Individuals with a total leukocyte count above 50 × 10^9^/l were advised to contact their general practitioner for further evaluation. The study was approved by a Danish ethical committee (H-KF-01-144/01), and all participants gave written informed consent.

### Covariates

All included covariates were chosen based on previous studies reporting them to be associated with lymphopenia [7,22–24] and with risk of infection [25–27]. Information on age and sex was acquired from the Danish Civil Registration System, whereas information on smoking status (current/former/never), cumulative smoking in pack-years (with 1 pack-year defined as 20 cigarettes or equivalent smoked per day for a year), alcohol consumption (none/moderate/heavy, with heavy consumption defined as >168 g/week for men and >84 g/week for women, as recommended by the Danish Health Authority), body mass index (measured weight in kilograms divided by the measured height in meters squared, categorized into 6 groups: ≤18.5/18.6–25.0/25.1–30.0/30.1–35.0/35.1–40.0/>40.0), recent infection within the last 4 weeks before the date of examination (including acute fever, bronchitis, and/or urinary tract infection), and medication use (any medication use or no medication use, with vitamins and herbals regarded as no medication use) were derived from the questionnaire and physical examination [18]. The categorizations of quantitative variables were done using conventional cutoffs to ease interpretation.

As a marker of inflammation, plasma C-reactive protein level was measured at the date of examination using standard high-sensitivity hospital assays. Blood neutrophil count was measured as part of the white blood cell differential count.

### Blood lymphocyte count

Blood samples were collected in EDTA tubes at the date of examination, and white blood cell counts were measured on fresh samples shortly after blood draw using the ADVIA 120 Hematology System. Precision of the measurements was monitored by daily measurements on internal control samples, while accuracy was monitored monthly using external control samples from a Scandinavian quality control program. The day-to-day coefficient of variation of the internal control was typically 2%–4% in the range of 25 × 10^9^/l.

Internationally, there is no consensus on the definition of lymphopenia. The cutoff value varies slightly between laboratories, but lymphopenia is commonly defined as a lymphocyte count below 1.0–1.5 × 10^9^/l [15,28,29]. In this study, we examined a general population cohort including both healthy and ill individuals, and we chose to categorize lymphocyte counts below the 2.5th percentile as lymphopenia (<1.1 × 10^9^/l), between the 2.5th and 97.5th percentile as the reference range (1.1–3.7 × 10^9^/l), and above the 97.5th percentile as lymphocytosis (>3.7 × 10^9^/l).

### Infectious disease endpoints

Using the Danish National Patient Register [30] which covers all Danish hospitals, we obtained information for each individual on hospitalizations and/or emergency room visits with a primary discharge diagnosis of an infectious disease. We collected this information until November 14, 2014. Information on emigration (*n =* 385) and vital status and date of emigration/death until November 14, 2014, was retrieved from the Danish Civil Registration System.

For individuals who died before November 14, 2014, information on infection-related deaths was obtained from the national Danish Register of Causes of Death [31], which covers all deaths in Denmark and registers all listed diagnoses on the death certificate as contributors to death according to the treating physician.

Infectious disease diagnoses were classified according to the World Health Organization’s International Statistical Classification of Diseases–10th Revision (ICD-10), and collapsed into the following 7 categories: pneumonia, skin infection, urinary tract infection, sepsis, diarrheal disease, endocarditis, and other infections (Table A in S1 Appendix). Of note, hospitalizations due to HIV/AIDS were not included in any of the infectious disease categories, since a low lymphocyte count in combination with a high risk of infection is characteristic for this disease. To investigate whether the Danish National Patient Register records infectious disease diagnoses correctly, a medical doctor specializing in infectious diseases validated 141 admissions coded as infections in the registry, and 139 of 141 admissions fulfilled the following criteria: (1) relevant signs and symptoms of infection, (2) a positive culture, and/or (3) treatment with antibiotics [32].

### Comorbidities

Noninfectious comorbidities could possibly confound the association between lymphopenia and risk of infection, since various malignancies and systemic autoimmune diseases and their respective treatments are associated with both a high risk of infection and a low lymphocyte count [9,14,15,33,34]. We therefore adjusted for the Charlson comorbidity index [35], a severity-weighted index of comorbid conditions that considers both the number and severity of comorbid diseases. This index has been validated for its ability to predict mortality [36,37]. We retrieved information from the Danish National Patient Register on inpatient hospitalizations, emergency room visits, and outpatient visits regarding the following disease categories: AIDS/HIV, any malignancy (including lymphoma and leukemia, except malignant neoplasms of the skin), cerebrovascular disease, chronic pulmonary disease, congestive heart failure, dementia, diabetes without chronic complications, diabetes with chronic complications, hemiplegia/paraplegia, metastatic solid tumors, mild liver disease, moderate/severe liver disease, myocardial infarction, peptic ulcer disease, peripheral vascular disease, renal disease, and rheumatic disease, using ICD-10 codes previously published [37], and as previously done [18]. This was done to reduce the possible confounding effect of comorbidities on the association between lymphopenia and risk of infection and infection-related death.

To exclude that the observed association was due to individuals having immunodeficiency/hematologic disease or autoimmune disease [38], information on diagnoses of immunodeficiencies, hematologic diseases, and autoimmune diseases was retrieved from the Danish National Patient Register using the ICD-10 codes shown in Tables B and C in S1 Appendix. Individuals with HIV infection were categorized as being immunodeficient and included in all main analyses.

### Statistical analyses

Statistical analyses were performed using Stata version 13.1. All statistical tests were 2-sided, and *p-*values < 0.05 were considered statistically significant. Jointly among all authors before study initiation, analyses were planned and adjustment variables were decided upon before analyses were conducted. The only change to the initial analysis plan was the inclusion of the Charlson comorbidity index and information on immunodeficiency/hematologic disease, to account for potential confounders associated with both lymphocyte count and risk of infection. Also, comments from reviewers prompted additional sensitivity analyses to be conducted and information on autoimmune diseases to be included in the multivariable adjusted model. Differences in baseline characteristics were assessed using Cuzick’s extension to the Wilcoxon rank-sum test for continuous variables and logistic regression for categorical variables. Correlations between lymphocyte count and covariates were calculated using Spearman’s rank correlation.

**Fig 2:**
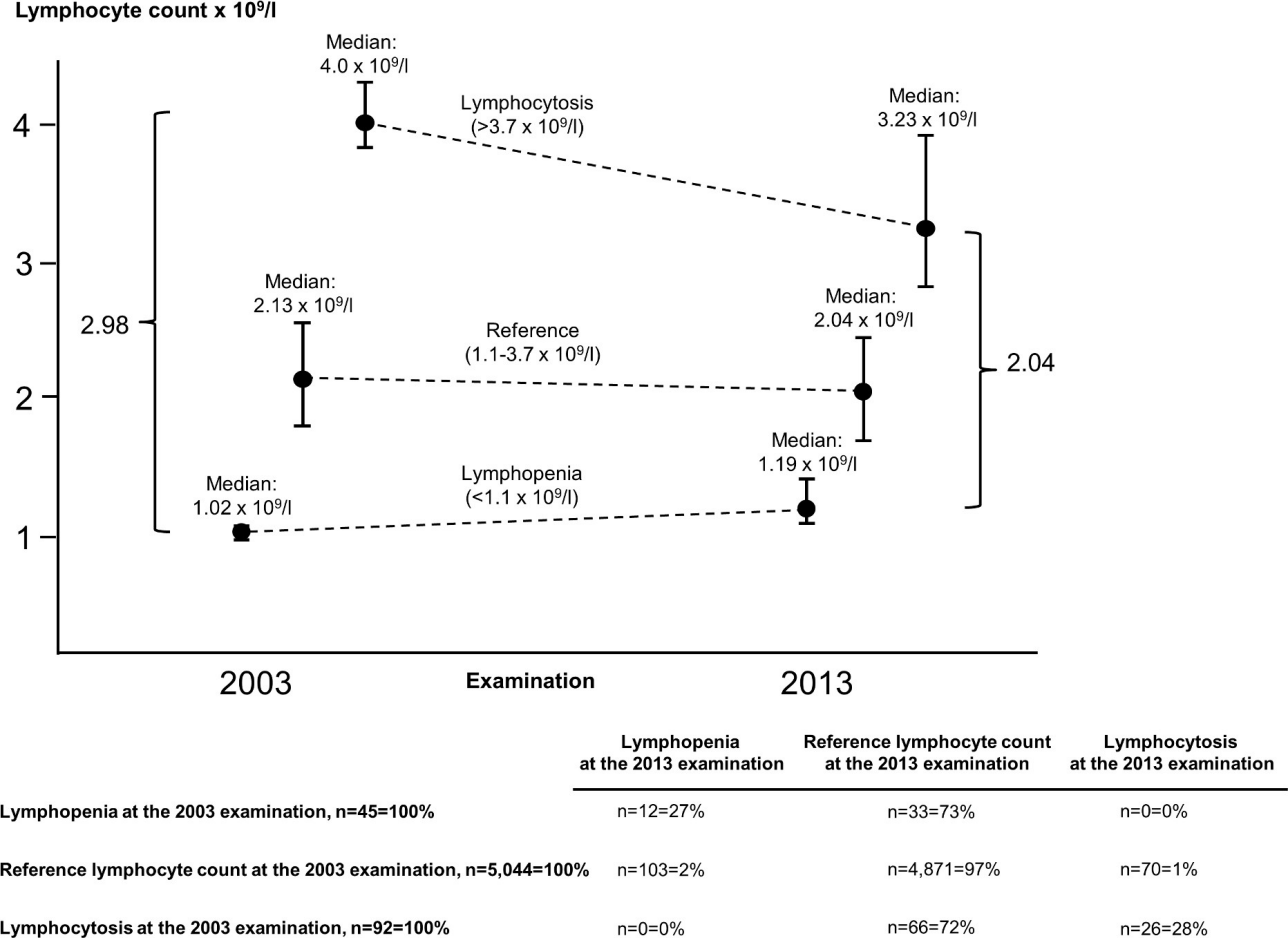
Regression toward the mean of lymphocyte counts, categorized as lymphopenia (<1.1 × 10^9^/l), reference lymphocyte count (1.1–3.7 × 10^9^/l), and lymphocytosis (>3.7 × 10^9^/l) at the date of examination in 2003 in the Copenhagen General Population Study. When categorizing lymphocyte counts, lymphopenia was defined as a lymphocyte count below the 2.5th percentile, the reference category was defined as a lymphocyte count between the 2.5th and 97.5th percentile, and lymphocytosis was defined as a lymphocyte count above the 97.5th percentile. Median values and interquartile ranges of 5,181 individuals at the 2003 examination (left) and the same individuals—maintaining the 2003 categorization—at the 2013 examination (right). Regression dilution ratio is 2.04/2.98 = 0.68. The table below the graph gives the percentage of individuals in each lymphocyte category at the 2003 examination (rows) and at the 2013 examination (columns).

To estimate the stability of blood lymphocyte counts, we calculated regression toward the mean using the lymphocyte count at the initial date of examination (the 2003 examination, from 2003–2004) and the lymphocyte count after a median of 10.5 years (the 2013 examination, from 2013–2014) in 5,181 individuals who had repeat measurements performed. The regression dilution ratio was calculated according to Clarke et al. [39]. In our study, the ratio was based on the median value in the groups with lymphopenia and lymphocytosis, using the 2.5th and 97.5th percentile cutoffs for lymphocytes at the date of examination, and was calculated to 0.68 (Fig 2). This analysis was not planned before first analyses, but performed after first results were known.

Risk of infectious disease hospitalization and risk of infection-related death were modeled separately using Cox proportional hazards regression with left-truncated age as the timescale, implying that age is automatically adjusted for. For presentation of results from the Cox models, hazard ratios and 95% confidence intervals according to continuous levels of lymphocyte counts were modeled using restricted cubic splines. The number of knots for the splines, between 3 and 7, was chosen according to which number of knots had the lowest value of Akaike’s information criterion [40]; if Akaike’s information criterion values were within 2 of each other for different numbers of knots, the lowest number of knots was chosen. In this study, all cubic splines are presented with 5 knots. Risk estimates are also presented with lymphocyte counts categorized into 3 groups: lymphopenia was defined as a lymphocyte count below the 2.5th percentile, the reference category was defined as a lymphocyte count between the 2.5th and 97.5th percentile, and lymphocytosis was defined as a lymphocyte count above the 97.5th percentile. For the analysis of lymphocyte count and risk of infectious disease hospitalization, follow-up began at the date of examination and ended on the date of infectious disease hospitalization, death due to any cause (*n =* 5,636), emigration (*n =* 385), or November 14, 2014, whichever came first. For the analysis of lymphocyte count and risk of infection-related death, follow-up began at the date of examination and ended on date of infection-related death, death due to another cause, emigration, or November 14, 2014, whichever came first. Interactions were tested for using a likelihood ratio test. These analyses were planned before initiation of the study.

Apart from age as the underlying timescale, multivariable models were additionally adjusted for sex and values at baseline of smoking status, cumulative smoking in pack-years, alcohol consumption, body mass index, plasma C-reactive protein, blood neutrophil count, recent infection, Charlson comorbidity index, autoimmune diseases, medication use, and immunodeficiency/hematologic disease. These adjustments were planned before initiation of the study, except for the inclusion of the Charlson comorbidity index and autoimmune diseases, which were added to the model at a later stage.

Information on age, sex, blood neutrophil count, Charlson comorbidity index, autoimmune diseases, and immunodeficiency/hematologic disease was available on all individuals. For the remaining covariates, information was more than 98% complete. Missing data were coded as missing for the categorical variables smoking status, alcohol consumption, body mass index, recent infection, and medication use, while missing data for the continuous variables cumulative smoking and plasma C-reactive protein were imputed based on age, sex, and, for cumulative smoking, smoking status using linear regression. However, when only individuals with complete information on all covariates were included, results were similar to those reported.

### Sensitivity analyses

Hypothetically, an association between lymphopenia and increased risk of future infection could be due to subclinical infections present, but not yet diagnosed, at the time of blood sampling. To test the robustness of the association, we therefore subdivided follow-up time into the following 3 time intervals after blood sampling: 0–2 years, more than 2 and up to 4 years, and more than 4 years. This analysis was prespecified, as done earlier [18].

Furthermore, we examined whether comorbidities diagnosed after the date of examination could explain the association between lymphopenia and risk of infection, accounting for any comorbidity undiagnosed at the date of examination. This sensitivity analysis was performed by including the Charlson comorbidity index as a time-dependent variable in the multivariable adjusted model, using continuously updated information on comorbidities during follow-up retrieved from the Danish National Patient Register. This analysis was not planned before first analyses, but performed after first results were known.

Individuals infected with HIV/AIDS could potentially confound our analyses, since these individuals have low lymphocyte counts in combination with a high risk of infection. To test the impact of this confounder, we conducted a sensitivity analysis excluding all individuals diagnosed with HIV/AIDS at date of examination or during follow-up (*n =* 12). This analysis was not planned before first analyses, but prompted by editors’ comments.

### Clinical relevance of lymphopenia for predicting risk of infection in individual persons

To predict an individual’s risk of future hospitalization due to infection, we performed receiver operating characteristic curve analyses, evaluating the sensitivity and specificity of different blood lymphocyte cutoffs to predict hospitalization due to infection within 2 years of blood sampling. The analyses were done using at first only the lymphocyte count and then the lymphocyte count taking age, sex, and smoking status into account. To further help clinicians in their evaluation of individuals with lymphopenia, we made a prediction algorithm for absolute 2-year risk of hospitalization due to infection, stratified according to other infectious disease risk factors. Inclusion of other infectious disease risk factors in the prediction algorithm was based on the multivariable adjusted model, as we only included risk factors with a significant interaction test in the prediction algorithm. To estimate absolute 2-year risk of infectious disease hospitalization, we used Poisson regression models. Importantly, all individuals with immunodeficiency, hematologic disease, and/or autoimmune disease were not included in the algorithm or the receiver operating characteristic curve analyses, since these individuals have a well-known high risk of infection. These analyses were not planned before first analyses, but prompted by reviewers’ comments.

## Results

Baseline characteristics recorded at the date of examination for 98,344 individuals from the Copenhagen General Population Study are shown in Table 1. All characteristics were independently associated with risk of infection. Lymphocyte counts are presented in Fig A in S1 Appendix. Lymphopenia was defined as a lymphocyte count below the 2.5th percentile of the population distribution (<1.1 × 10^9^/l).

**Table 1:** Baseline characteristics of 98,344 individuals from the Copenhagen General Population Study according to blood lymphocyte count.

Characteristic	Blood lymphocyte count			*p* for trend^a^	Correlation with blood lymphocyte count	
	Lymphopenia (<1.1 × 10^9^/l)	Reference (1.1–3.7 × 10^9^/l)	Lymphocytosis (>3.7 × 10^9^/l)		*r*	*p*
Individuals, number (%)	2,352 (2.4)	93,538 (95.1)	2,454 (2.5)	—	—	—
Blood lymphocyte count, ×10^9^/l (range)	1.0 (0.1–1.1)	2.1 (1.1–3.7)	4.0 (3.7–125.2)	—	—	—
Age, years (IQR)	68 (58–77)	58 (48–67)	57 (48–66)	2 × 10^−75^	−0.16	<1 × 10^−300^
Male sex, number (%)	1,303 (55)	41,957 (45)	921 (38)	2 × 10^−30^	—	—
Ever smokers, number (%)	1,364 (58)	53,972 (58)	1,842 (75)	2 × 10^−50^	—	—
Cumulative smoking, pack-years^b^ (IQR)	16 (5–33)	15 (6–30)	26 (14–40)	6 × 10^−104^	0.12	<1 × 10^−300^
Alcohol consumption >168/84 g/week^c^, number (%)	955 (41)	36,803 (39)	884 (36)	0.01	—	—
Body mass index, kg/m^2^ (IQR)	25 (23–28)	26 (23–28)	27 (24–30)	4 × 10^−54^	0.12	<1 × 10^−300^
Plasma C-reactive protein, mg/l (IQR)	1.6 (1.0–3.4)	1.4 (1.0–2.2)	1.9 (1.3–3.4)	3 × 10^−32^	0.09	4 × 10^−170^
Blood neutrophil count, × 10^9^/l (IQR)	3.7 (2.9–4.8)	4.0 (3.3–4.9)	5.0 (4.1–6.1)	3 × 10^−244^	0.21	<1 × 10^−300^
Any recent infection, number (%)	131 (6)	3,658 (4)	131 (5)	0.63	—	—
Any comorbidity^d^, number (%)	1,114 (47)	18,892 (20)	565 (23)	5 × 10^−41^	—	—
Charlson score 1 or 2	808 (34)	16,262 (17)	485 (20)	2 × 10^−21^	—	—
Charlson score > 2	306 (13)	2,639 (3)	80 (3)	2 × 10^−48^	—	—
Any autoimmune disease, number (%)	298 (13)	4,252 (5)	117 (5)	4 × 10^−19^	—	—
Any medication, number (%)	1,743 (74)	52,384 (56)	1,540 (63)	0.002	—	—
Any immunodeficiency/hematologic disease^f^, number (%)	250 (11)	2,220 (2)	130 (5)	1 × 10^−6^	—	—

Values are median for continuous variables and frequency for categorical variables. When categorizing lymphocyte counts, lymphopenia was defined as a lymphocyte count below the 2.5th percentile, the reference category was defined as a lymphocyte count between the 2.5th and 97.5th percentile, and lymphocytosis was defined as a lymphocyte count above the 97.5th percentile.

^a^*p* for trend was calculated with Cuzick’s extension of the Wilcoxon rank-sum test (continuous variables) or logistic regression (categorical variables). Correlations (*r*, and *p*-values) between covariates and lymphocyte count were calculated with Spearman’s correlation.

^b^Only ever smokers.

^c^>168 g/week for men and >84 g/week for women, following the Danish Health Authority.

^d^As defined by the Charlson comorbidity index.

^f^HIV/AIDS is included in the immunodeficiency category.

Among 5,181 individuals with blood lymphocyte counts at both the 2003 and 2013 examination, 27% of those who had lymphopenia at the 2003 examination still had it at the 2013 examination (Fig 2). The median blood lymphocyte count in individuals with lymphopenia was 1.02 × 10^9^/l (IQR 0.96–1.06) at the 2003 examination, while the corresponding value in the same individuals measured 10 years later at the 2013 examination was 1.19 × 10^9^/l (IQR 1.08–1.40). These values together with the values for individuals with lymphocytosis gave a regression dilution ratio of 0.68 (2.04/2.98; see Fig 2). Notably, 84% of those who had lymphopenia at the 2003 examination had lymphocyte count < 1.5 × 10^9^/l at the 2013 examination. Likewise important, among individuals without lymphopenia at the 2003 examination, 2% had lymphopenia at the 2013 examination.

### Lymphopenia and risk of infection

**Fig 3:**
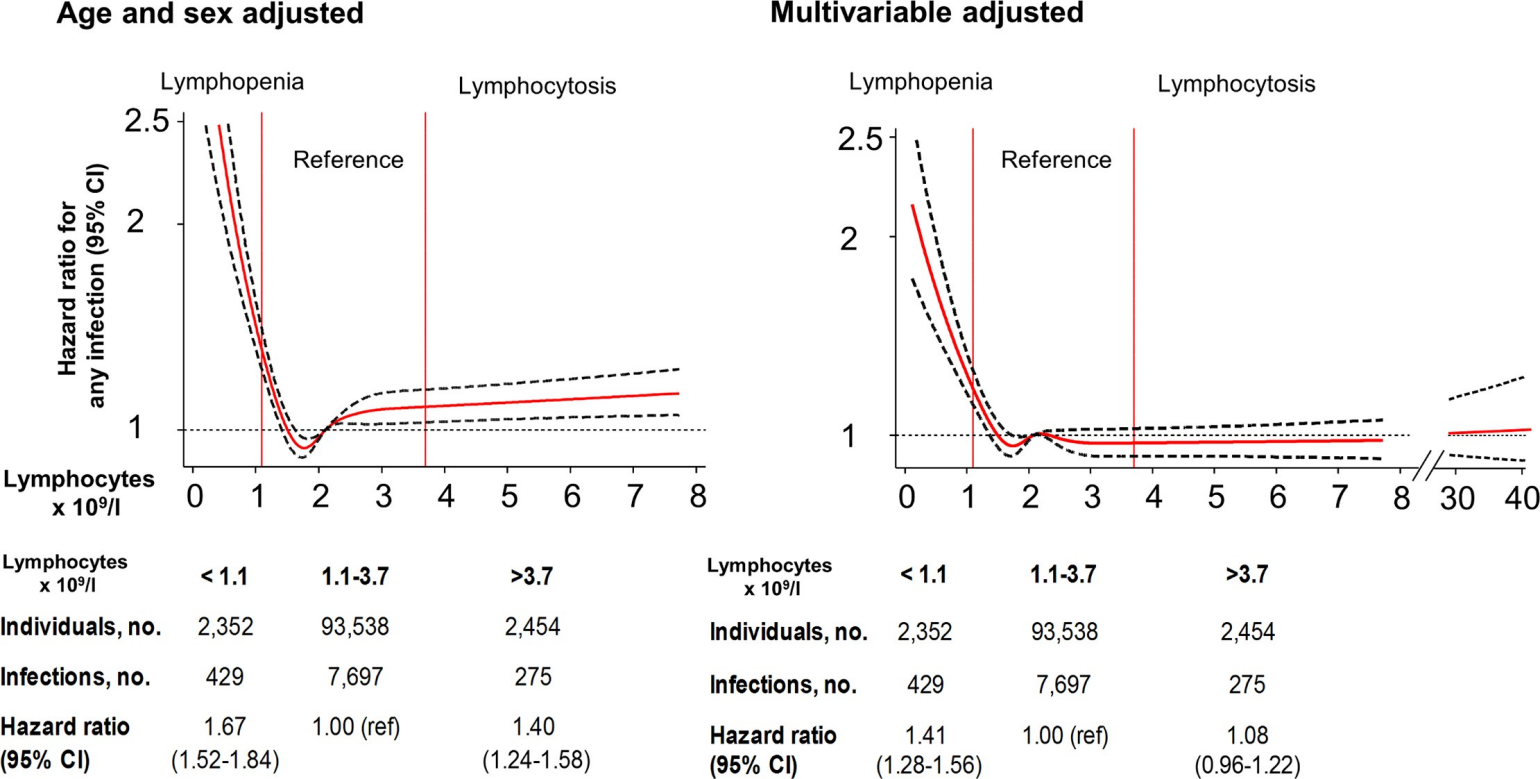
Risk of any infection as a function of lymphocyte count for individuals from the Copenhagen General Population Study. Solid red lines are hazard ratios, and dashed black lines indicate 95% confidence intervals based on fitting of cubic splines to risk estimates obtained using Cox proportional hazards regression. Multivariable adjustment includes all covariates listed in Table 1 except age, but with age as the underlying timescale. The median lymphocyte value of 2.1 × 10^9^/l was set as reference for the continuous model. For the multivariable adjusted model, the lymphocyte interval 30–40 × 10^9^/l is added to illustrate that for very high lymphocyte counts the risk of infections increases, but with a broad confidence interval. When categorizing lymphocyte counts, lymphopenia was defined as a lymphocyte count below the 2.5th percentile, the reference category was defined as a lymphocyte count between the 2.5th and 97.5th percentile, and lymphocytosis was defined as a lymphocyte count above the 97.5th percentile.

During a median follow-up of 6 years (range 0–11 years), 8,401 individuals were hospitalized due to infection. After adjusting for age and sex, the hazard ratio for any infection was 1.67 (95% CI 1.52–1.84) in individuals with lymphopenia and 1.40 (95% CI 1.24–1.58) in individuals with lymphocytosis, when compared to individuals with lymphocytes in the reference range (Fig 3). In the multivariable adjusted model, corresponding hazard ratios were 1.41 (95% CI 1.28–1.56) and 1.08 (95% CI 0.96–1.22).

The spline curves in Fig 3 illustrate the change in hazard ratios and 95% confidence intervals for risk of any infection as a function of blood lymphocyte count on a continuous scale. The reference value, i.e., where the hazard ratio is set to 1.0, is the median lymphocyte count for the entire population (2.1 × 10^9^/l), and smoothed curves that best fit the data are shown. Fig 3 shows that at blood lymphocyte counts below 1.4 × 10^9^/l, the multivariable adjusted hazard ratio for any infection increases sharply with lower and lower lymphocyte counts, ultimately reaching 2.16 at lymphocyte counts of 0.1 × 10^9^/l. At blood lymphocyte counts above the median value, the hazard ratio for any infection did not differ from 1.0 in the multivariable adjusted model.

**Fig 4:**
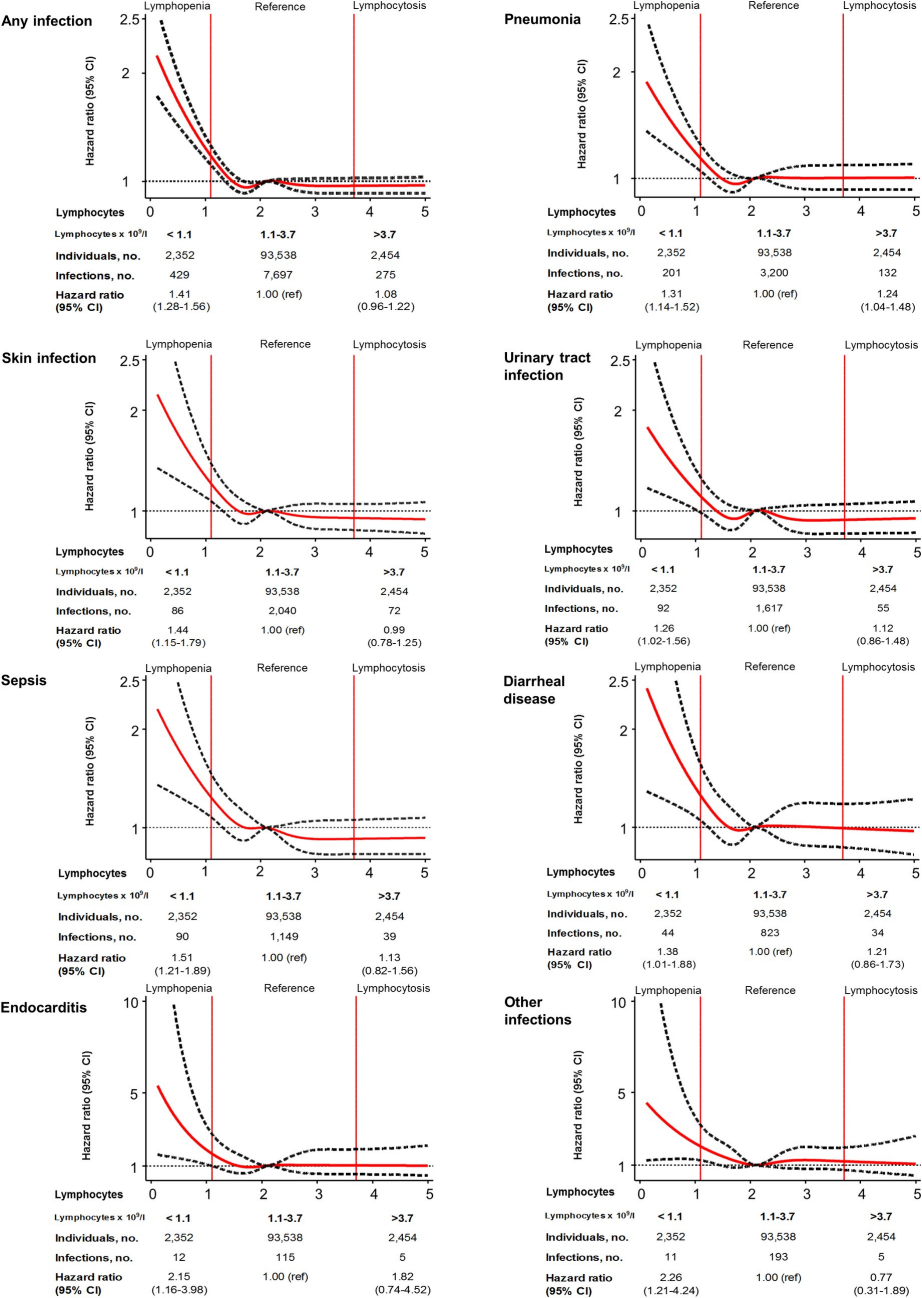
Multivariable adjusted risks of specific infections as a function of lymphocyte count for individuals from the Copenhagen General Population Study. Solid red lines are multivariable adjusted hazard ratios, and dashed black lines indicate 95% confidence intervals based on fitting of cubic splines to risk estimates obtained using Cox proportional hazards regression. Multivariable adjustment includes all covariates listed in Table 1 except age, but with age as the underlying timescale. The median lymphocyte value of 2.1 × 10^9^/l was set as reference for the continuous model. The sum of the numbers of cases of specific infections exceeds the number of cases of “any infection” since individuals could have more than 1 specific infection. When categorizing lymphocyte counts, lymphopenia was defined as a lymphocyte count below the 2.5th percentile, the reference category was defined as a lymphocyte count between the 2.5th and 97.5th percentile, and lymphocytosis was defined as a lymphocyte count above the 97.5th percentile.

For specific types of infections, individuals with lymphopenia versus those with lymphocytes in the reference range had multivariable adjusted hazard ratios (95% CIs) of 1.31 (1.14–1.52) for pneumonia, 1.44 (1.15–1.79) for skin infection, 1.26 (1.02–1.56) for urinary tract infection, 1.51 (1.21–1.89) for sepsis, 1.38 (1.01–1.88) for diarrheal disease, 2.15 (1.16–3.98) for endocarditis, and 2.26 (1.21–4.24) for other infections (Fig 4).

Results were similar to those presented in Fig 4 if lymphopenia was defined using other commonly applied international cutoffs for lymphopenia (<1.0 or <1.5 × 10^9^/l) (Figs B and C in S1 Appendix). When adjusting the analysis for regression dilution bias, we found a multivariable adjusted hazard ratio of 1.65 (95% CI 1.43–1.91) for any infection in individuals with lymphopenia compared to individuals with lymphocytes in the reference range (Fig D in S1 Appendix).

### Stratified analyses

**Fig 5:**
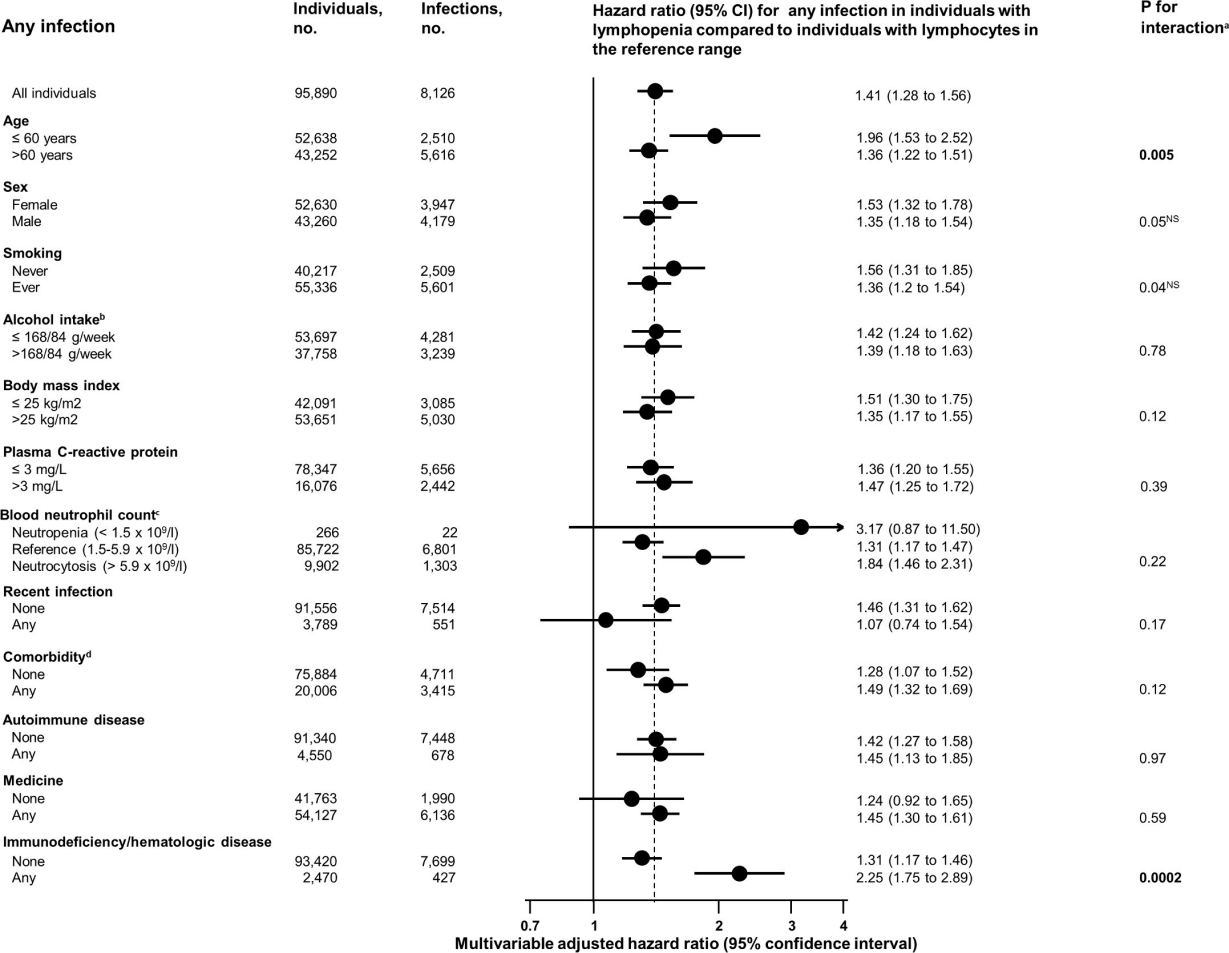
Multivariable adjusted risk of any infection for individuals from the Copenhagen General Population Study with lymphopenia (lymphocyte count < 1.1 × 10^9^/l) compared to individuals with lymphocytes in the reference range (1.1–3.7 × 10^9^/l). Multivariable adjustment includes all covariates listed in Table 1 except age, but with age as the underlying timescale. The sum number of individuals in the strata varies slightly because of varying numbers of individuals with missing data in the covariates. When categorizing lymphocyte counts, lymphopenia was defined as a lymphocyte count below the 2.5th percentile, while the reference category was defined as a lymphocyte count between the 2.5th and 97.5th percentile. ^NS^Not significant after adjusting for 12 multiple comparisons using the Bonferroni method.^a^According to a likelihood ratio test.^b^≤168 versus >168 g/week for men and ≤84 versus >84 g/week for women.^c^When categorizing neutrophil counts, neutropenia was defined as a neutrophil count below 1.5 × 10^9^/l, which is the generally accepted lower cutoff [4,41–43], the reference category was defined as a neutrophil count from 1.5 × 10^9^/l to 5.9 × 10^9^/l, and neutrocytosis was defined as a neutrophil count above 5.9 × 10^9^/l.^d^As defined by the Charlson comorbidity index.

**Fig 6:**
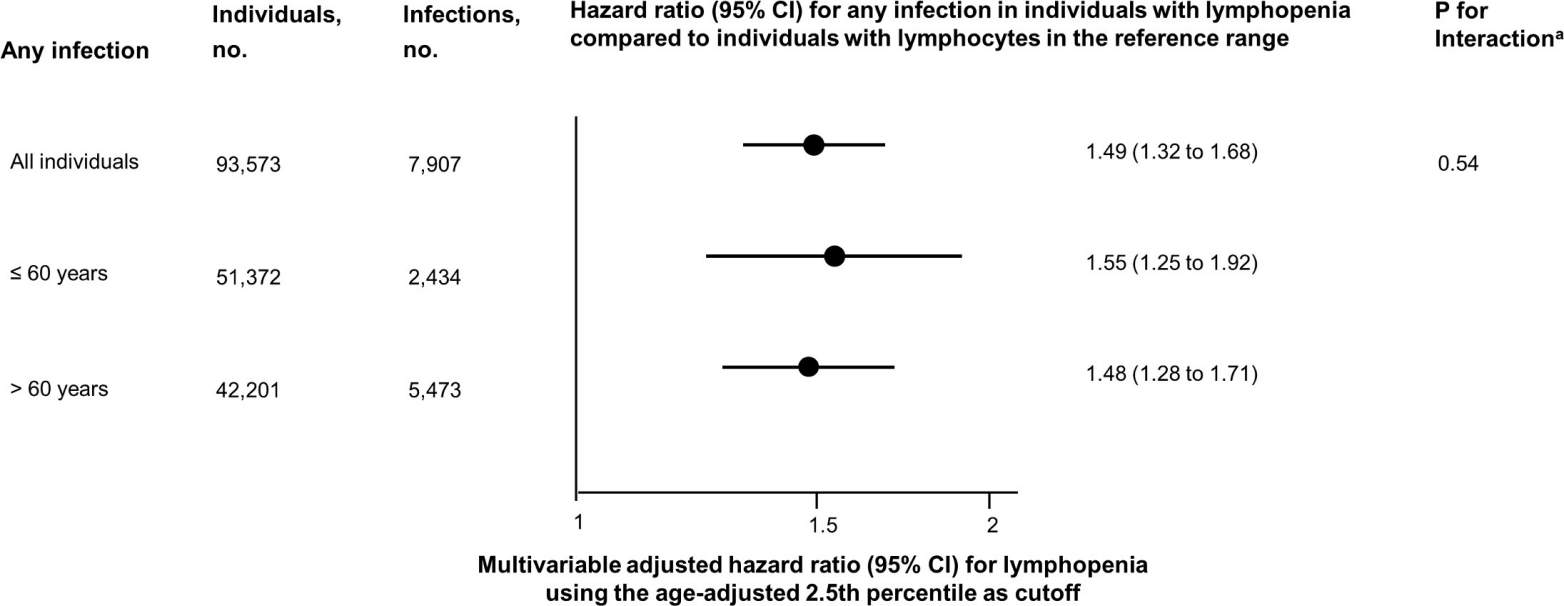
Multivariable adjusted risk of any infection for individuals from the Copenhagen General Population Study with lymphopenia using age-adjusted percentiles of lymphocyte count. Multivariable adjusted risk of any infection for individuals from the Copenhagen General Population Study with lymphopenia (lymphocyte count < 1.1 × 10^9^/l) compared to individuals with lymphocytes in the reference range (1.1–3.7 × 10^9^/l). Multivariable adjustment includes all covariates listed in Table 1 except age, but with age as the underlying timescale. Individuals with lymphopenia had a lymphocyte count below the 2.5th percentile in each 1-year age band. ^a^According to a likelihood ratio test.

To examine the robustness of the association between lymphopenia and risk of any infection, we performed stratified analyses according to strata of the covariates that were associated with lymphocyte counts at baseline (Fig 5). Risk of any infection in those with lymphopenia versus those with lymphocytes in the reference range was higher in individuals aged ≤60 years than in those aged >60 years (*p* for interaction = 0.005). Risk of any infection in those with lymphopenia versus those with lymphocytes in the reference range was higher in those with immunodeficiency/hematologic disease than in those without (*p* for interaction < 0.001) at date of examination. Importantly, however, risk of any infection in those with lymphopenia was still elevated in individuals aged >60 years and in those without immunodeficiency/hematologic disease. To further explore the interaction with age, we redefined lymphopenia by calculating the age-adjusted 2.5th percentile for lymphocyte count for each 1-year age span. When using these age-adjusted 2.5th percentiles as the cutoff for lymphopenia, lymphopenia was still associated with increased risk of any infection, with a hazard ratio of 1.49 (95% CI 1.32–1.68), but there was no longer an interaction between age and lymphopenia (*p* = 0.54) on risk of any infection (Fig 6).

### Sensitivity analyses

**Fig 7:**
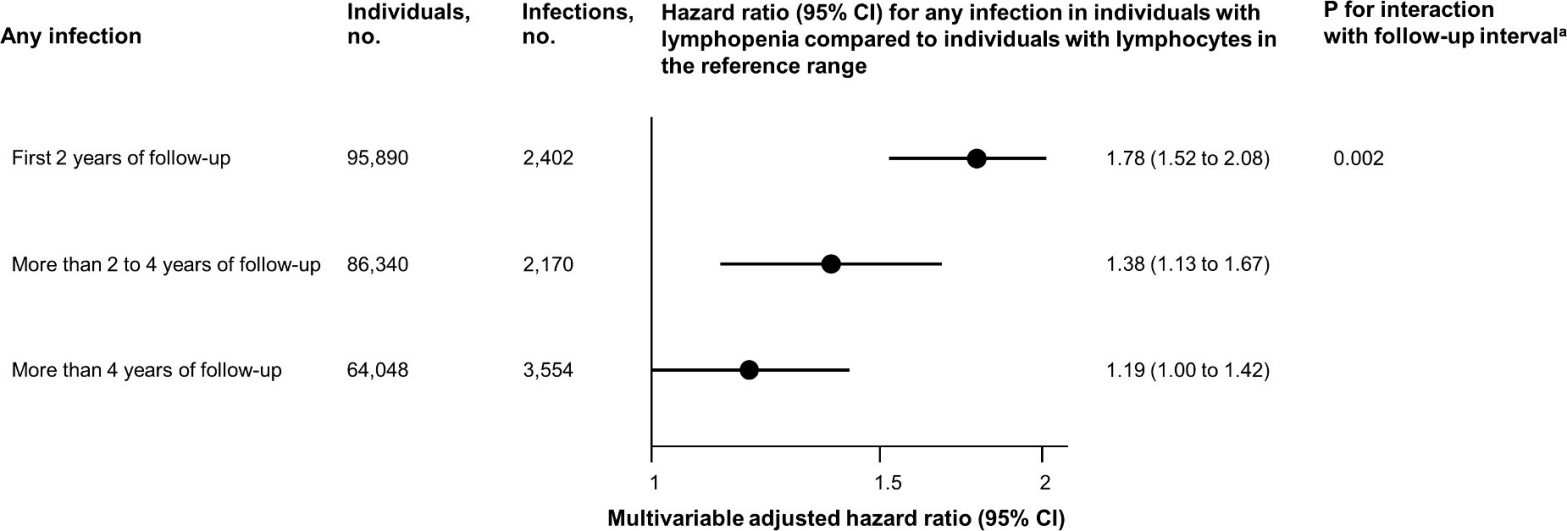
Multivariable adjusted risk of any infection for individuals from the Copenhagen General Population Study with lymphopenia stratified by follow-up time. Multivariable adjusted risk of any infection for individuals from the Copenhagen General Population Study with lymphopenia (lymphocyte count < 1.1 × 10^9^/l) compared to individuals with lymphocyte count in the reference range (1.1–3.7 × 10^9^/l). Multivariable adjustment includes all covariates listed in Table 1 except age, but with age as the underlying timescale. Follow-up time was segmented into 3 time intervals, and each individual was included in more than 1 time interval if total follow-up was above 2 years. When categorizing lymphocyte counts, lymphopenia was defined as a lymphocyte count below the 2.5th percentile, while the reference category was defined as a lymphocyte count between the 2.5th and 97.5th percentile. ^a^According to a likelihood ratio test.

To examine whether the observed association was due to reverse causation or heavily confounded by undiagnosed comorbidities at baseline, we subdivided follow-up time into the following 3 time intervals after blood sampling: 0–2 years, more than 2 and up to 4 years, and more than 4 years (Fig 7). Although the risk estimate was attenuated with increasing time from the date of examination (*p* for interaction = 0.002), risk was still increased more than 2 years after the date of examination and onwards. When taking comorbidities diagnosed after the date of examination into consideration, the association between lymphopenia and risk of any infection was only slightly attenuated (Fig E in S1 Appendix).

Of 98,344 individuals included, only 12 were infected with HIV/AIDS at the date of examination or during follow-up. When we excluded these individuals from the main multivariable adjusted analysis, results were similar (Fig F in S1 Appendix).

### Clinical relevance of lymphopenia for predicting risk of infection in individual persons

**Fig 8:**
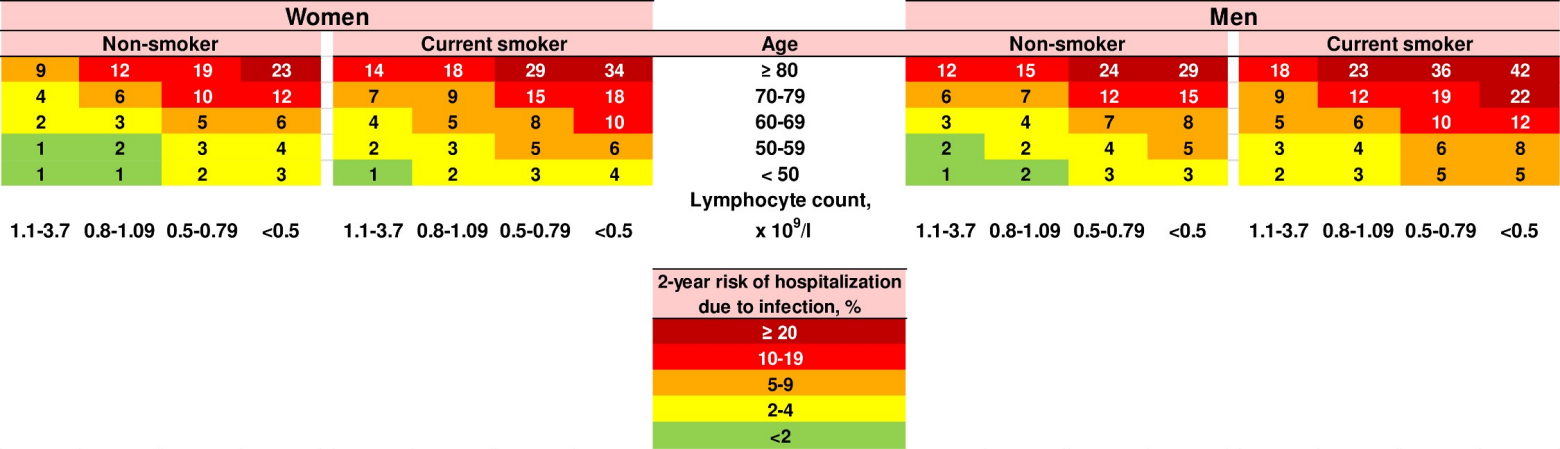
Algorithm. Absolute 2-year risks of hospitalization due to infection for different blood lymphocyte categories, stratified for age, sex, and smoking status. Nonsmokers were former smokers and never-smokers combined. Individuals diagnosed with immunodeficiency, hematologic disease, and/or autoimmune disease were not included when calculating absolute risks for the algorithm, since these individuals have a well-known high risk of infection. Numbers indicate absolute 2-year risk as rounded percentages.

To examine lymphopenia as a tool to predict an individual’s risk of future hospitalization due to infection, we performed receiver operating characteristic curve analyses, evaluating the sensitivity and specificity of different blood lymphocyte cutoffs to predict hospitalization due to infection within 2 years of blood sampling (Figs G and H in S1 Appendix). Generally, when using a low blood lymphocyte cutoff (i.e., 1.1 × 10^9^/l), the specificity was high, but the sensitivity was low, indicating that for very low lymphocyte counts, an individual was likely to be admitted to hospital due to an infection, but that a high proportion of infectious disease hospitalizations occurred in individuals without lymphopenia. As low blood lymphocyte count only explains a small proportion of infectious disease hospitalizations in the overall population, lymphocyte count alone could not convincingly predict risk of infection in most individuals, as illustrated by an area under the curve of 0.55 (95% CI 0.54–0.56) (Fig G in S1 Appendix), and when further taking age, sex, and smoking status into account, results were similar (Fig H in S1 Appendix). However, when we developed a prediction algorithm taking age, sex, and smoking status into account, we were able to differentiate individuals with high absolute 2-year risk of infection from those with very low risk (Fig 8). Based on inspiration from the Framingham Risk Score for predicting risk of cardiovascular disease, our algorithm predicts absolute 2-year risk of hospitalization due to any infection, taking lymphocyte count, age, sex, and smoking status into account. As seen in Fig 8, the combination of lymphopenia with other risk factors enables the algorithm to categorize individuals into categories with markedly different absolute 2-year risks of infection. For example, the highest absolute 2-year risk of 42% was observed for male smokers aged >80 years with blood lymphocyte count < 0.5 × 10^9^/l. This means that nearly 1 in 2 individuals from this category will be admitted to hospital due to an infectious disease within 2 years of the date of examination. In contrast, the lowest absolute 2-year risk was observed for nonsmokers aged <50 years with lymphocyte count > 0.8 × 10^9^/l, irrespective of sex.

### Lymphopenia and infection-related death

**Fig 9:**
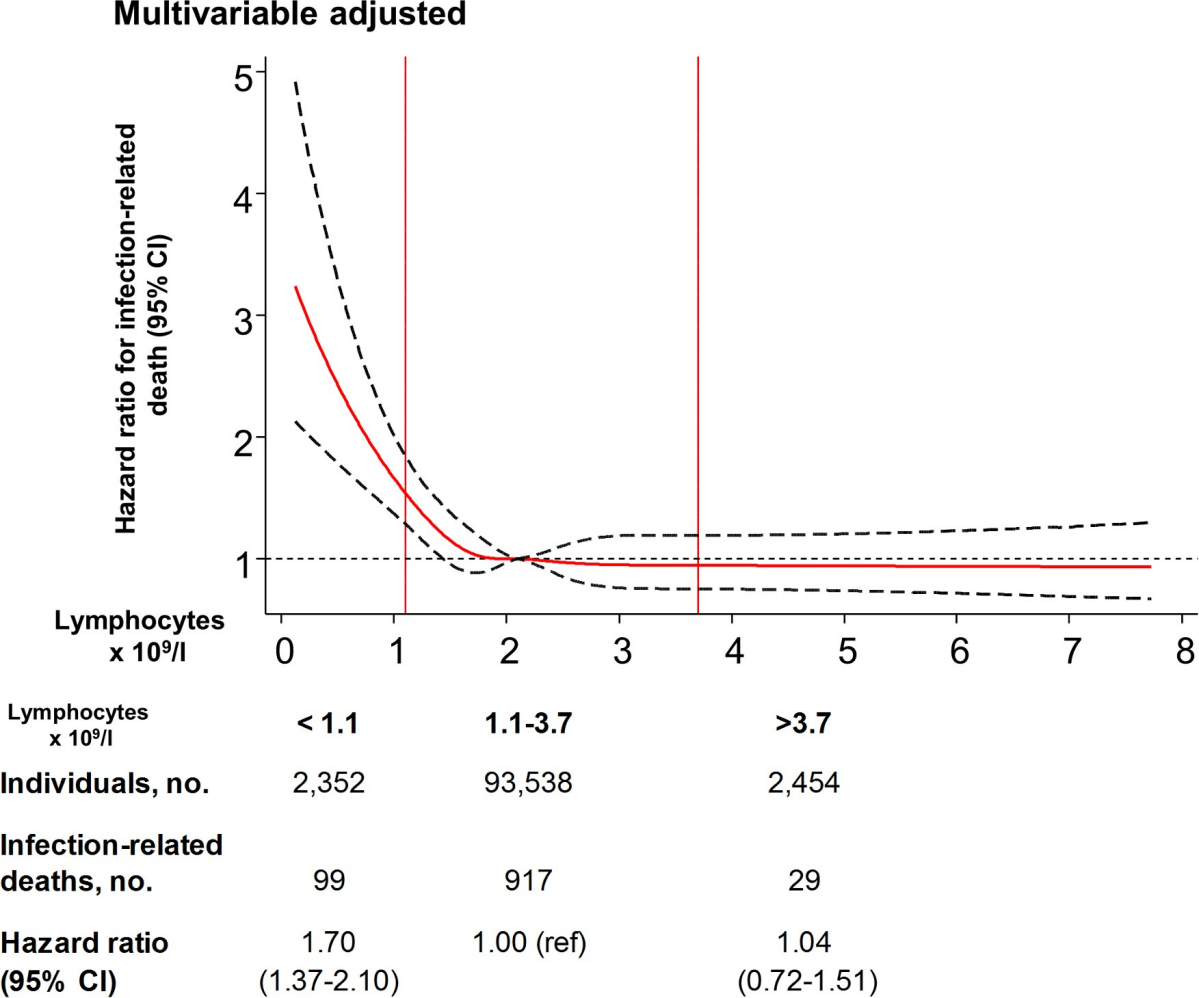
Multivariable adjusted risk of infection-related death as a function of lymphocyte count for individuals from the Copenhagen General Population study. The solid red line is the multivariable adjusted hazard ratio, and black dashed lines indicate the 95% confidence interval based on fitting of cubic splines to risk estimates obtained using Cox proportional hazards regression. Multivariable adjustment includes all covariates listed in Table 1 except age, but with age as the underlying timescale. The median lymphocyte value of 2.1 × 10^9^/l was set as reference for the continuous model. When categorizing lymphocyte counts, lymphopenia was defined as a lymphocyte count below the 2.5th percentile, the reference category was defined as a lymphocyte count between the 2.5th and 97.5th percentile, and lymphocytosis was defined as a lymphocyte count above the 97.5th percentile.

The hazard ratio for infection-related death in individuals with lymphopenia versus those with lymphocytes in the reference range was 1.70 (95% CI 1.37–2.10) in the multivariable adjusted model (Fig 9).

## Discussion

In this prospective study of 98,344 individuals from the general population, we found that lymphopenia was associated with increased risk of hospitalization due to any infection, pneumonia, skin infection, urinary tract infection, sepsis, diarrheal disease, endocarditis, and other infections after adjusting for potential explanatory factors including blood neutrophil count. Lymphopenia was also associated with a 1.7-fold increased risk of infection-related death. These are novel findings. However, causality cannot be deduced from our data.

### Comparison with other studies

There are surprisingly few studies on lymphopenia and risk of infection, and none of them have studied individuals in the general population. One study found a 2.4-fold increased risk of urinary tract infection and lower respiratory tract infection among 156 long-term facility care residents with lymphopenia [33], and 2 studies with 167 and 91 participants found that lymphopenia in patients with systemic autoimmune diseases was associated with a 5.2- and 4.7-fold increased risk of infection, respectively [14,15]. Further, a recent study examining 753 patients in intensive care units found that lymphopenia at admittance was associated with a 1.6-fold increased risk of infection, but there was no association between lymphopenia and 28-day mortality. However, persistent lymphopenia at day 3 after admittance was associated with both a 1.4-fold increased risk of infection and a 1.7-fold increased risk of 28-day mortality [44]. Discrepancies between the findings in the present and former studies [14,15,33] may be due to our study design including general population individuals, versus case cohorts of fragile or very sick individuals/patients. Also, as former studies were smaller than the present one, effect sizes in previous studies may have needed to be larger than what we observed for the results to be significant at the *p* < 0.05 level. Hence, due to potential publication bias, there may have been a tendency for previous smaller studies to be published only if they reported large effect sizes, while similar smaller studies with negative results may never have been published. Such a scenario is well known in the medical literature, i.e., that the first relatively small studies typically report larger effect sizes than later-published large or huge studies.

### Strengths and limitations

Strengths of this study include the large study cohort and prospective design. Also, the 98,344 individuals from the general population with a blood lymphocyte count were followed without losses to follow-up due to the unique Danish civil and health registers. Furthermore, we were able to adjust for various potentially explanatory factors such as smoking, body mass index, alcohol intake, plasma C-reactive protein, blood neutrophil count, recent infection, medication use, and comorbidities including autoimmune disease and immunodeficiency/hematologic disease.

Potential limitations of our study should also be considered. This study is strictly observational and can therefore not address questions of causality, and our analyses might theoretically have been affected by reverse causation or confounding. However, since the association between lymphopenia and risk of infection was observed even for any infection occurring more than 2 years after the blood lymphocyte count, reverse causation is less likely to be the only explanation of the results, although the attenuation after 2 years of follow-up might be a sign of underlying conditions at time of examination. Confounding by lifestyle factors or other yet unknown factors is also possible. In the multivariable adjusted analyses, we adjusted for variation in known risk factors for infection; however, we naturally cannot exclude that other known or unknown confounders could explain the association between lymphopenia and risk of infection. Causality could be addressed in a randomized trial aimed at increasing lymphocyte counts; however, at present, such a trial is not feasible. Alternatively, a genetic Mendelian randomization study could address causality; however, we are not aware of sufficiently strong genetic instruments to address this question in a study the size of ours. Importantly, from a clinical point of view, if the observed association is due to individuals with lymphopenia actually having an undiagnosed comorbidity, this does not make the motivation to follow these individuals less relevant.

Another potential limitation could be that individuals with lymphopenia were more likely to be admitted to hospital by their general practitioner or the hospital staff than individuals with lymphocytes in the reference range. However, for individuals with lymphopenia, the estimates for risk of infection and risk of infection-related death were similar. Thus, we found no indication that the severity of infection in patients with lymphopenia is reduced compared to that in patients with lymphocytes in the reference range.

Stability of blood lymphocyte counts over the course of the study also needs to be addressed. Using repeat measurements 10 years apart of blood lymphocyte counts in 5,181 individuals, we found that even though lymphocyte counts vary over time, most individuals with lymphopenia at the date of the initial examination had lymphocyte counts below 1.5 × 10^9^/l 10 years later. Conversely, some individuals moved from one lymphocyte category to another after 10 years, and this could contribute to the attenuation of risk estimates observed with longer follow-up periods. Regression dilution bias considers both measurement and biological variability over time, and analyses adjusted for regression dilution bias gave more pronounced results, but did not change the overall results.

Another potential bias is healthy participant bias because the 45% of invited individuals who did participate were likely healthier than the non-participants. If that was the case, then the observed association between lymphopenia and increased risk of infection possibly could be underestimated. Lastly, the generalizability of our results to other populations may be reduced by the fact that we only investigated white individuals of Danish descent; however, we are not aware of data indicating that the results presented in this paper may be different in other ethnic groups. Indeed, blood lymphocyte counts seem similar in different ethnicities [45,46]. In the present study we did not include race as a variable for adjustment, as all individuals included were white and of Danish descent.

### Possible explanations and implications for clinicians

Circulating lymphocytes are 70%–80% composed of T lymphocytes, implying that lymphopenia is most likely a manifestation of a reduced number of T lymphocytes [13]. Circulating lymphocytes are a part of the extensive defense against microorganisms in the body, and lymphocytes can be very long-living cells with memory functions, resulting in a quick recognition and eradication of microorganisms known to the immune system. Lymphocyte counts decrease with increasing age, as also shown here, and the composition of lymphocyte subsets changes as individuals age [47]. Other important age-related immune changes are the impaired generation of primary CD8^+^ T cell responses against infections and reduced vaccine efficacy [48–50]. Furthermore, older age is characterized by thymic involution and a decline in naïve T and B cell output from the bone marrow, which leads to accumulation of memory T cells that are late differentiated or senescent, with the majority specific for chronic virus infections such as cytomegalovirus or Epstein-Barr virus [51]. It is well known that the risk of infection increases with higher age, probably due to both acquired comorbidities and change in overall immune competence, including alterations in lymphocyte subsets [52]. Previous studies have shown that the composition and kinetics of T lymphocyte subsets predict infections [53], and in the setting of HIV and AIDS, it is well established that a low CD4^+^ cell count is a major risk factor for opportunistic infections. Likewise, a decrease in T cell receptor diversity has been shown in HIV-infected individuals [54], indicating that T cell receptor diversity might be dependent on the number and composition of circulating lymphocytes. Furthermore, T cell receptor signal transduction pathways are significantly altered with age, resulting in impaired transcription of T cell genes [55]. In our study of the general population, we did not measure specific lymphocyte subsets; thus, we do not know to what degree lymphopenia was pronounced in each subset of lymphocytes. Nonetheless, based on the abovementioned studies, it is plausible that lymphopenia in individuals from the general population may cause a decrease in the T cell receptor repertoire, which might theoretically explain why these individuals experience a greater risk of infection.

Thus, an association between a low lymphocyte count and increased risk of infection physiologically seems plausible, especially in aged individuals, but age alone cannot explain our findings since all analyses were adjusted for age, and the analysis using age-specific cutoffs for lymphopenia produced similar results.

In this study, we defined lymphopenia as a lymphocyte count below the 2.5th percentile in the study population, and when using 2 widely implemented cutoffs—1.0 × 10^9^/l and 1.5 × 10^9^/l—results were similar, indicating that this is a robust finding.

Today doctors primarily evaluate only neutropenia in individuals from the general population, due to the well-known high risk of infection [3]. A review from 2012 recommended screening individuals with neutrophil counts < 1.0 × 10^9^/l for active or chronic infection with viruses, bacteria, mycobacteria, and rickettsia; investigating possible drug-induced or autoimmune neutropenia; and performing a bone marrow biopsy for neutrophil counts < 0.5 × 10^9^/l [4]. For lymphocyte counts < 1.0 × 10^9^/l, the same review recommended screening for HIV and measurement of immunoglobulins, but no further evaluation if these tests are normal. Thus, physicians are currently not recommended to intervene in individuals with “asymptomatic” lymphopenia without an associated diagnosed disease. To evaluate whether lymphopenia may be useful to clinicians for predicting an individual’s absolute risk of future infection, we developed a prediction algorithm to determine absolute 2-year risk of hospitalization due to any infection in individuals from the general population with low lymphocyte counts. The 2-year risk estimates vary from 1% to 42%, with the highest risk observed in older male smokers with lymphocyte counts < 0.5 × 10^9^/l. Our algorithm indicates that prevention or early detection of infections in individuals with lymphopenia may be especially relevant to implement for individuals who have other concurrent risk factors (e.g., male sex, high age, smoking), as these individuals can have a very high absolute risk of infectious disease hospitalization. Hence, the usefulness of combining several risk factors resembles that of the well-known Framingham Risk Score for prediction and prevention of cardiovascular disease [56]. The results from the present study could indicate that individuals with lymphopenia might benefit from additional medical attention because of their increased risk of infection as well as increased risk of infection-related death.

### Conclusion

Of the invited 220,424 individuals, 99,191 attended examination. In the 98,344 individuals from the general population with a valid lymphocyte count, lymphopenia, defined as blood lymphocytes < 1.1 × 10^9^/l, was associated with a 1.4-fold increased risk of later hospitalization with infection and a 1.7-fold increased risk of infection-related death. Importantly, causality cannot be deduced from our data.

## Supporting information

S1 Appendix Supplementary tables (A–C) and figures (A–H).
(DOCX)

S1 STROBE Checklist Checklist for reporting of observational studies in epidemiology. (DOCX)

S1 Text The Copenhagen General Population Study questionnaire. (PDF)

S2 Text Medication questionnaire. (PDF)
