# Supplementary material for: Lymphopenia and risk of infection and infection-related death in 98,344 individuals from a prospective Danish population-based study
Source: PLoS Med. 2018 Nov 1;15(11):e1002685. doi: 10.1371/journal.pmed.1002685 (PMC6211632; doi:10.1371/journal.pmed.1002685)
Supplement: S1 Appendix — Supplementary tables (A–C) and figures (A–H). (DOCX) [file pmed.1002685.s001.docx]

**Lymphopenia and risk of infection and infection-related death in 98,344 individuals from a prospective Danish population-based study**

**S1 Appendix**

Marie Warny^1,2^, Jens Helby^3,4^, Børge Grønne Nordestgaard^2,3,4^, Henrik Birgens^1,2^ and Stig Egil Bojesen^2,3,4^

^1^Department of Hematology, Herlev and Gentofte Hospital, Copenhagen University Hospital, DK-2730 Herlev, Denmark

^2^Faculty of Health and Medical Science, University of Copenhagen, DK-2200 Copenhagen, Denmark

^3^Department of Clinical Biochemistry, Herlev and Gentofte Hospital, Copenhagen University Hospital, DK-2730 Herlev, Denmark

^4^Copenhagen General Population Study, Herlev and Gentofte Hospital, Copenhagen University Hospital, DK-2730 Herlev, Denmark

**Table of contents Page No.**

Table A: Categorization of infectious diseases according to the World Health 3

Organization's International Statistical Classification of Diseases, revision 10 (ICD-10)

Table B: Classification of hematologic disease and immunodeficiency disease 4

according to the World Health Organization's International Statistical Classification of Diseases,

revision 10 (ICD-10)

Table C: Classification of autoimmune disease according to the World Health 5

Organization's International Statistical Classification of Diseases, revision 10 (ICD-10)

Fig A: The distribution of lymphocyte counts in the Copenhagen General Population Study 6

Fig B: Multivariable adjusted risks of specific infections as a function of lymphocyte count 7-8

for individuals from the Copenhagen General Population Study, with lymphopenia defined as a

lymphocyte count < 1.0 x 10^9^/l

Fig C: Multivariable adjusted risks of specific infections as a function of lymphocyte count 9-10

for individuals from the Copenhagen General Population Study, with lymphopenia defined as a

lymphocyte count < 1.5 x 10^9^/l

Fig D: Risk of any infection as a function of lymphocyte count for individuals from 11

the Copenhagen General Population Study, after adjusting for regression dilution ratio

Fig E: Time-dependent Charlson Comorbidity Index 12

Fig F: Risk of any infection as a function of lymphocyte count for individuals from the 13

Copenhagen General Population Study, after exclusion of individuals infected with HIV/AIDS

Fig G: Receiver operating characteristic curve of sensitivity as a function of 1-specificity for cutoffs 14

of lymphocyte counts to predict individual risk of future hospitalization due to an infection.

Fig H: Receiver operating characteristic curve of sensitivity as a function of 1-specificity for cutoffs 15

of lymphocyte counts, adjusted for age, sex and smoking status, to predict individual risk of future

hospitalization due to an infection.

References 16

| **Table A: Categorization of infectious diseases according to the World Health Organization's International Statistical Classification of Diseases, revision 10 (ICD-10)** | | | | |
| --- | --- | --- | --- | --- |
| **Disease category** |  | **Number of infections** |  | **ICD-10 codes** |
|  |  |  |  |  |
| **Pneumonia** |  | 3,533 |  | A481, J13-J16, J170, J18 |
|  |  |  |  |  |
| **Skin Infection** |  | 2,198 |  | A46, L00-L08, L303, L308F |
|  |  |  |  |  |
| **Urinary Tract Infection** |  | 1,764 |  | N109A-N109C, N110-N118B, |
|  |  |  |  | N118D, N119, N12, N300, |
|  |  |  |  | N308A-N308C, N309, N390 |
|  |  |  |  |  |
| **Sepsis** |  | 1,278 |  | A021, A282B, A327, A392-A394, |
|  |  |  |  | A40-A41, A427, A483, A499A, R572 |
|  |  |  |  |  |
| **Diarrheal disease** |  | 901 |  | A020, A022-A029, A03-A05, A08-A09 |
|  |  |  |  |  |
| **Endocarditis** |  | 132 |  | I33, I38, I398 |
|  |  |  |  |  |
| **Other infections** |  | 209 |  |  |
| Meningitis |  |  |  | A390, A87, B003, B004A, |
|  |  |  |  | G00-G01, G020, G039, G042 |
|  |  |  |  |  |
| Mycosis |  |  |  | B35-B49 |
|  |  |  |  |  |
| Hepatitis |  |  |  | B15-B19, Z225 |
|  |  |  |  |  |
| Imported and parasitic infections |  |  |  | A00-A01, A06-A07, A90-A96, B50-B64 |
|  |  |  |  |  |
| Influenza and viral lower |  |  |  | J09-J101C, J12, J171 |
| respiratory tract infection |  |  |  |  |
|  |  |  |  |  |
| Tuberculosis |  |  |  | A15-A19, N330, N740-N741 |
|  |  |  |  |  |
| Parasitic worm disease |  |  |  | B65-B83, N308J |
|  |  |  |  |  |
| Pertussis |  |  |  | A37 |
| Infectious disease categories are ranked according to the number of events in each category (highest to lowest). | | | | |

| **Table B: Classification of hematologic disease and immunodeficiency** | | | | | | | |  |
| --- | --- | --- | --- | --- | --- | --- | --- | --- |
| **disease according to the World Health Organization's International Statistical** | | | | | | |  |  |
| **Classification of Diseases, revision 10 (ICD-10)** | | | | |  |  |  |  |
| **Disease category** |  | **Number of individuals** |  | **Number of individuals** |  | **ICD-10 codes** | |  |
|  |  |  |  | **with any infection** |  |  |  |  |
|  |  |  |  |  |  |  |  |  |
| **Hematologic disease** |  | 2,371 |  | 428 |  | C77 |  |  |
|  |  |  |  |  |  | C81-C96 |  |  |
|  |  |  |  |  |  | D45-D47 |  |  |
|  |  |  |  |  |  | D50-D53 |  |  |
|  |  |  |  |  |  | D55-D77 |  |  |
|  |  |  |  |  |  | L99 |  |  |
|  |  |  |  |  |  | M36 |  |  |
|  |  |  |  |  |  | N16 |  |  |
|  |  |  |  |  |  | R59 |  |  |
|  |  |  |  |  |  | R70-R72 |  |  |
|  |  |  |  |  |  | R89 |  |  |
|  |  |  |  |  |  |  |  |  |
| **Immunodeficiency disease** |  | 268 |  | 36 |  | D80-D89 |  |  |
|  |  |  |  |  |  | R75-R77 |  |  |
|  |  |  |  |  |  | B20-B24 |  |  |
|  |  |  |  |  |  | Z21 |  |  |
|  |  |  |  |  |  | F024 |  |  |
|  |  |  |  |  |  |  |  |  |

| **Table C: Classification of autoimmune disease according to the World Health Organization's International Statistical Classification of Diseases, revision 10 (ICD-10)** | | | | | | |
| --- | --- | --- | --- | --- | --- | --- |
| **Disease category** |  | **Number of individuals** |  | **Number of individuals** |  | **IDC-10 codes** |
|  |  |  |  | **with any infection** |  |  |
|  |  |  |  |  |  |  |
| **Autoimmune disease** |  | 4,667 |  | 703 |  |  |
| Pernicious anemia |  |  |  |  |  | D51.0 |
| Autoimmune hemolytic anemia |  |  |  |  |  | D59.1 |
| Idiopathic thrombocytopenic purpura | | |  |  |  | D69.3 |
| Thyrotoxicosis |  |  |  |  |  | E05.0 |
| Autoimmune thyroiditis |  |  |  |  |  | E06.3 |
| Type 1 diabetes |  |  |  |  |  | E10 |
| Primary adrenocortical insufficiency | | |  |  |  | E27.1 |
| Multiple sclerosis |  |  |  |  |  | G35 |
| Guillain-Barre´ syndrome |  |  |  |  |  | G61.0 |
| Iridocyclitis |  |  |  |  |  | H20 |
| Crohn's disease |  |  |  |  |  | K50 |
| Ulcerative colitis |  |  |  |  |  | K51 |
| Autoimmune hepatitis |  |  |  |  |  | K73 |
| Primary biliary cirrhosis |  |  |  |  |  | K74.3 |
| Celiac disease |  |  |  |  |  | K90.0 |
| Pemphigus |  |  |  |  |  | L10 |
| Pemphigoid |  |  |  |  |  | L12 |
| Psoriasis vulgaris |  |  |  |  |  | L40 |
| Alopecia areata |  |  |  |  |  | L63 |
| Vitiligo |  |  |  |  |  | L80.9 |
| Seropositive rheumatoid arthritis |  |  |  |  |  | M05-M06 |
| Juvenile arthritis |  |  |  |  |  | M08 |
| Wegener's granulomatosis |  |  |  |  |  | M31.3 |
| Dermatopolymyositis |  |  |  |  |  | M33 |
| Polymyalgia rheumatica |  |  |  |  |  | M31.5-6, M35.3 |
| Myasthenia gravis |  |  |  |  |  | G70.0 |
| Scleroderma |  |  |  |  |  | M34 |
| Systemic lupus erythematosis |  |  |  |  |  | M32.1, M32.9 |
| Sjogren's syndrome |  |  |  |  |  | M35.0 |
| Ankylosing spondylitis |  |  |  |  |  | M45.9 |

Categorization of autoimmune diseases and ICD-10 codes are from the paper by Eaton et al.[1]





**Fig A: The distribution of lymphocyte counts in the Copenhagen General Population Study.** Solid lines indicate the 2.5th and 97.5th percentile. When categorizing lymphocyte counts, lymphopenia was defined as a lymphocyte count below the 2.5th percentile, the reference category was defined as a lymphocyte count between the 2.5th and 97.5th percentile, and lymphocytosis was defined as a lymphocyte count above the 97.5th percentile. The lymphocyte range is from 0.1 to 125.2 x 10^9^/l.


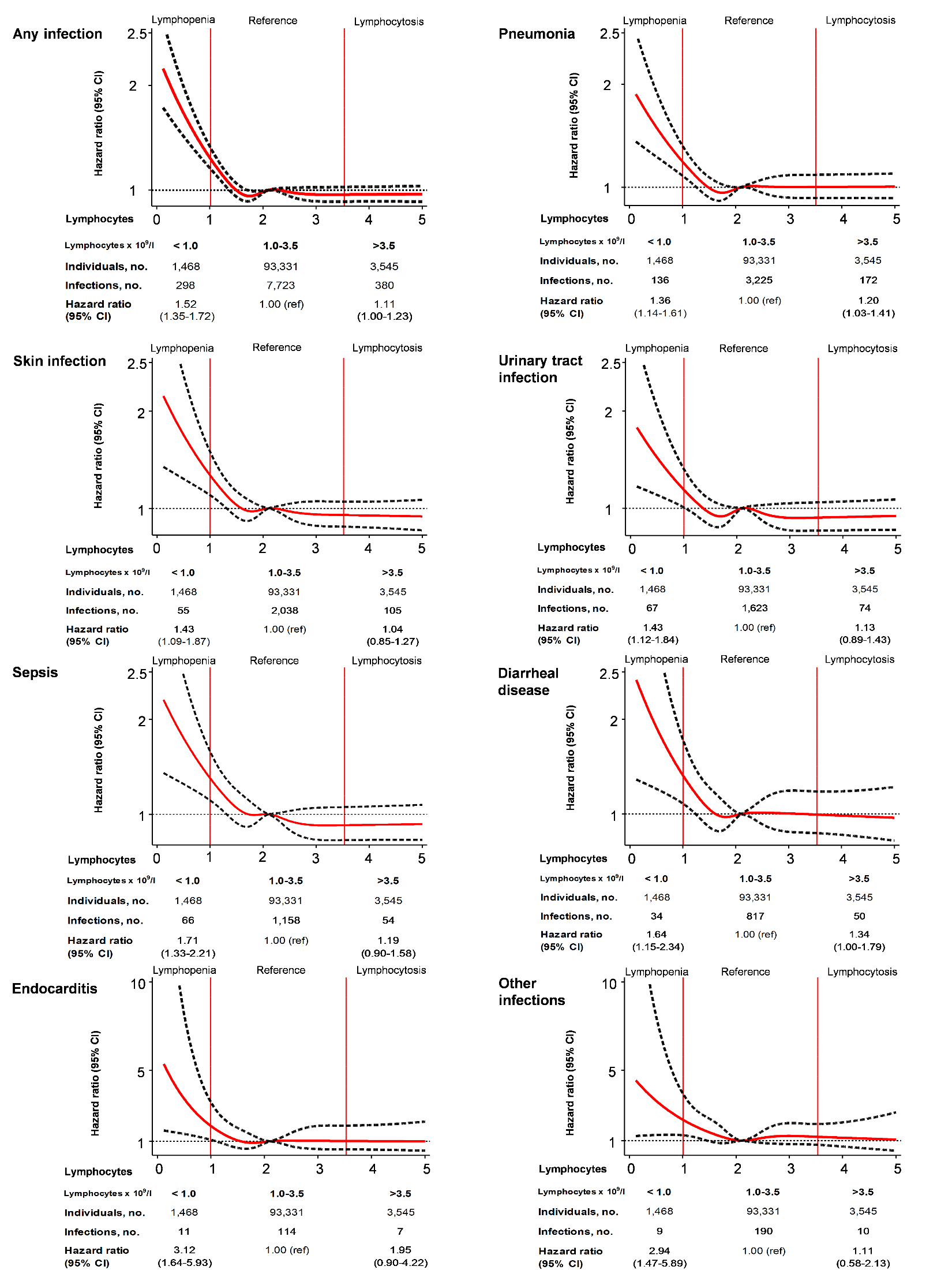


**Fig B: Multivariable adjusted risks of specific infections as a function of lymphocyte count for individuals from the Copenhagen General Population Study, with lymphopenia defined as a lymphocyte count < 1.0 x 10^9^/l.** Lymphopenia was defined as a lymphocyte count < 1.0 x 10^9^/l and lymphocytosis as a lymphocyte count > 3.5 x 10^9^/l. Solid red lines are multivariable adjusted hazard ratios, and dashed black lines indicate 95% confidence intervals based on fitting of cubic splines to risk estimates obtained using Cox proportional hazards regression. Multivariable adjustment includes all covariates listed in Table 1 except age, but with age as the underlying timescale. The median lymphocyte value of 2.1 x 10^9^/l was set as reference for the continuous model. The sum of the numbers of cases of specific infections exceeds the number of cases of “any infection” since individuals could have more than 1 specific infection. When categorizing lymphocyte counts, lymphopenia was defined as a lymphocyte count below the 2.5th percentile, the reference category was defined as a lymphocyte count between the 2.5th and 97.5th percentile, and lymphocytosis was defined as a lymphocyte count above the 97.5th percentile.

**
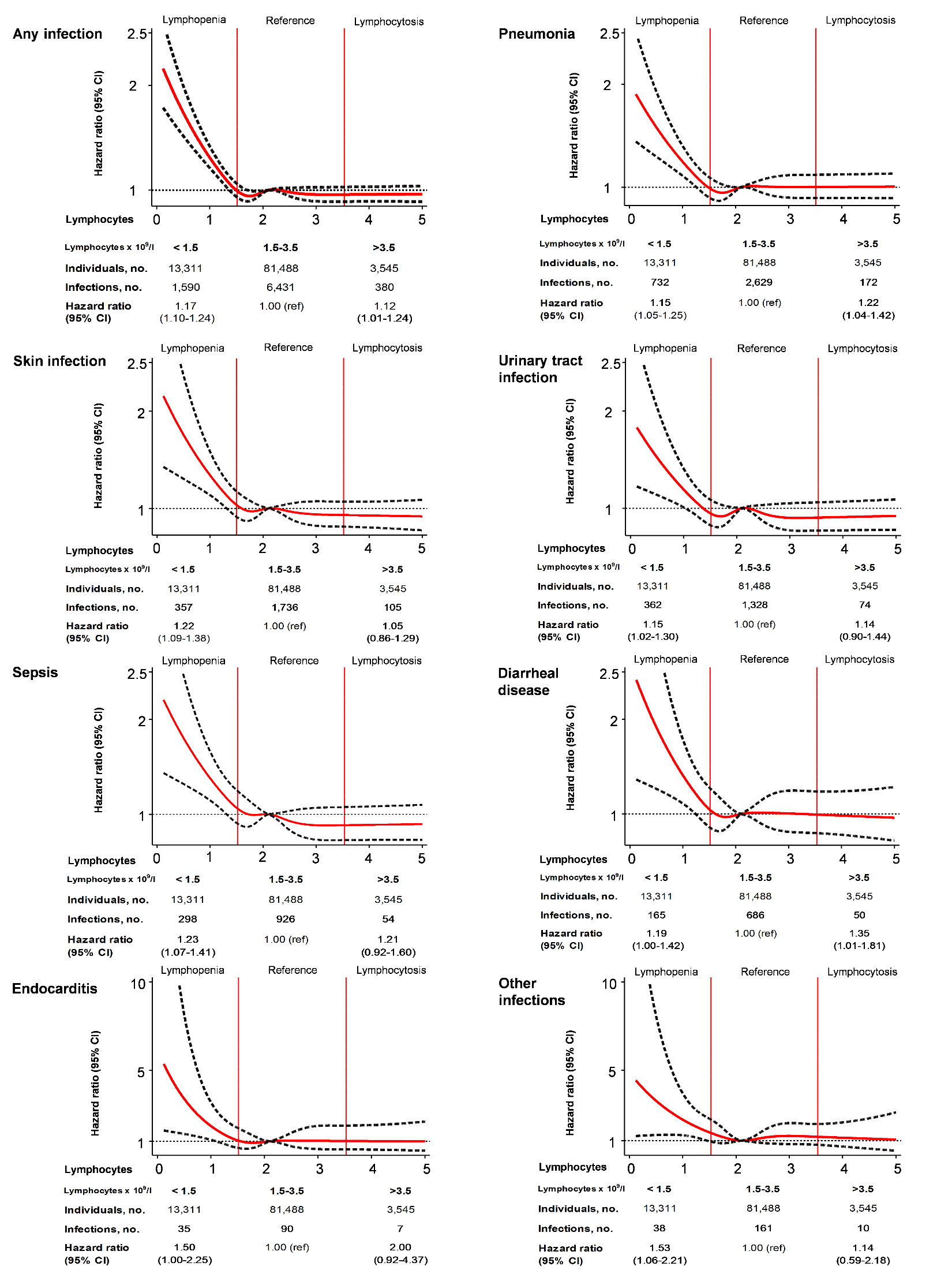
**

**Fig C: Multivariable adjusted risks of specific infections as a function of lymphocyte count for individuals from the Copenhagen General Population Study, with lymphopenia defined as a lymphocyte count < 1.5 x 10^9^/l.** Lymphopenia was defined as a lymphocyte count < 1.5 x 10^9^/l and lymphocytosis as a lymphocyte count > 3.5 x 10^9^/l. Solid red lines are multivariable adjusted hazard ratios, and dashed black lines indicate 95% confidence intervals based on fitting of cubic splines to risk estimates obtained using Cox proportional hazards regression. Multivariable adjustment includes all covariates listed in Table 1 except age, but with age as the underlying timescale. The median lymphocyte value of 2.1 x 10^9^/l was set as reference for the continuous model. The sum of the numbers of cases of specific infections exceeds the number of cases of “any infection” since individuals could have more than 1 specific infection. When categorizing lymphocyte counts, lymphopenia was defined as a lymphocyte count below the 2.5th percentile, the reference category was defined as a lymphocyte count between the 2.5th and 97.5th percentile, and lymphocytosis was defined as a lymphocyte count above the 97.5th percentile.

**
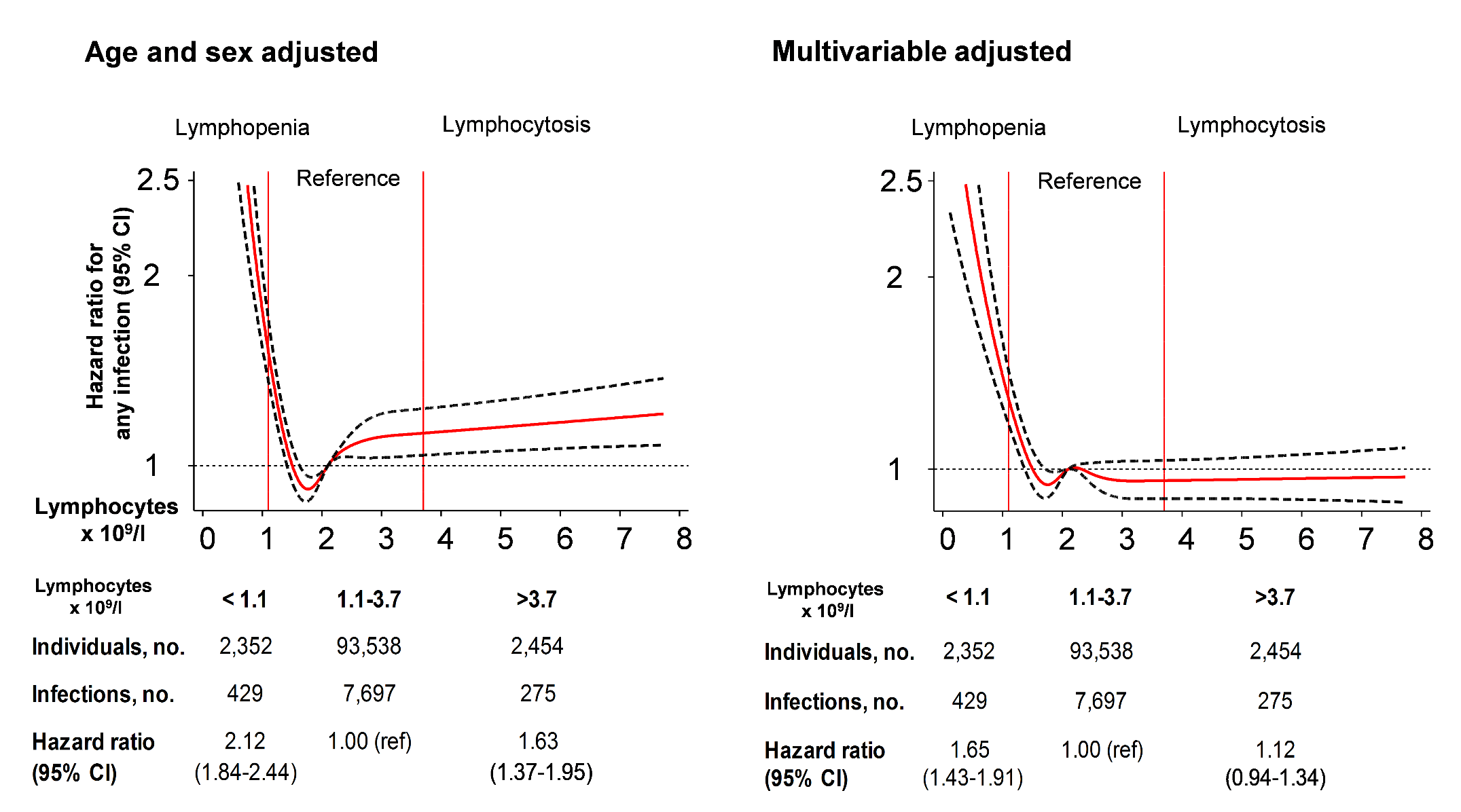
**

**Fig D: Risk of any infection as a function of lymphocyte count for individuals from the Copenhagen General Population Study, after adjusting for regression dilution ratio.** Solid red lines are hazard ratios, and dashed black lines indicate 95% confidence intervals based on fitting of cubic splines to risk estimates obtained using Cox proportional hazards regression. Multivariable adjustment includes all covariates listed in Table 1 except age, but with age as the underlying timescale. Results are furthermore adjusted for the regression dilution ratio using the lymphocyte count at the date of examination and the lymphocyte count after approximately 10 years in 5,181 individuals who had repeat measurements performed. The ratio was based on the median value when using the 2.5th and 97.5th percentiles of lymphocytes at baseline and was calculated to 0.68. The median lymphocyte value of 2.1 x 10^9^/l was set as reference for the continuous model. When categorizing lymphocyte counts, lymphopenia was defined as a lymphocyte count below the 2.5th percentile, the reference category was defined as a lymphocyte count between the 2.5th and 97.5th percentile, and lymphocytosis was defined as a lymphocyte count above the 97.5th percentile.

**
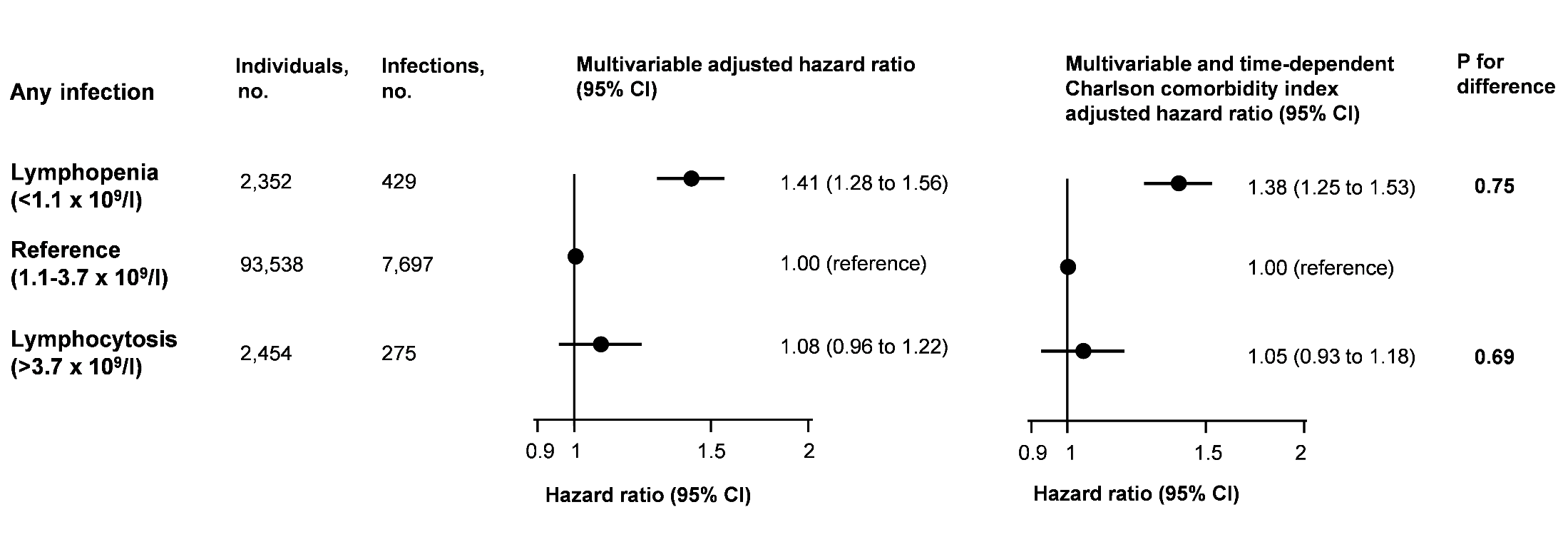
**

**Fig E: Time-dependent Charlson Comorbidity Index.** Multivariable adjusted risk of any infection for individuals from the Copenhagen General Population Study with lymphopenia (lymphocyte count <1.1 x 10^9^ /l) and lymphocytosis (>3.7 x 10^9^/l) compared to individuals with lymphocytes in the reference range (1.1-3.7 x 10^9^ /l). Multivariable adjustment includes all covariates listed in Table 1 except age, but with age as the underlying timescale (left) and the additional adjustment for the Charlson comorbidity index (right), as a time dependent variable, taking comorbidities diagnosed after the examination date into account. When categorizing lymphocyte counts, lymphopenia was defined as a lymphocyte count below the 2.5th percentile, the reference category was defined as a lymphocyte count between the 2.5th and 97.5th percentile, and lymphocytosis was defined as a lymphocyte count above the 97.5th percentile.


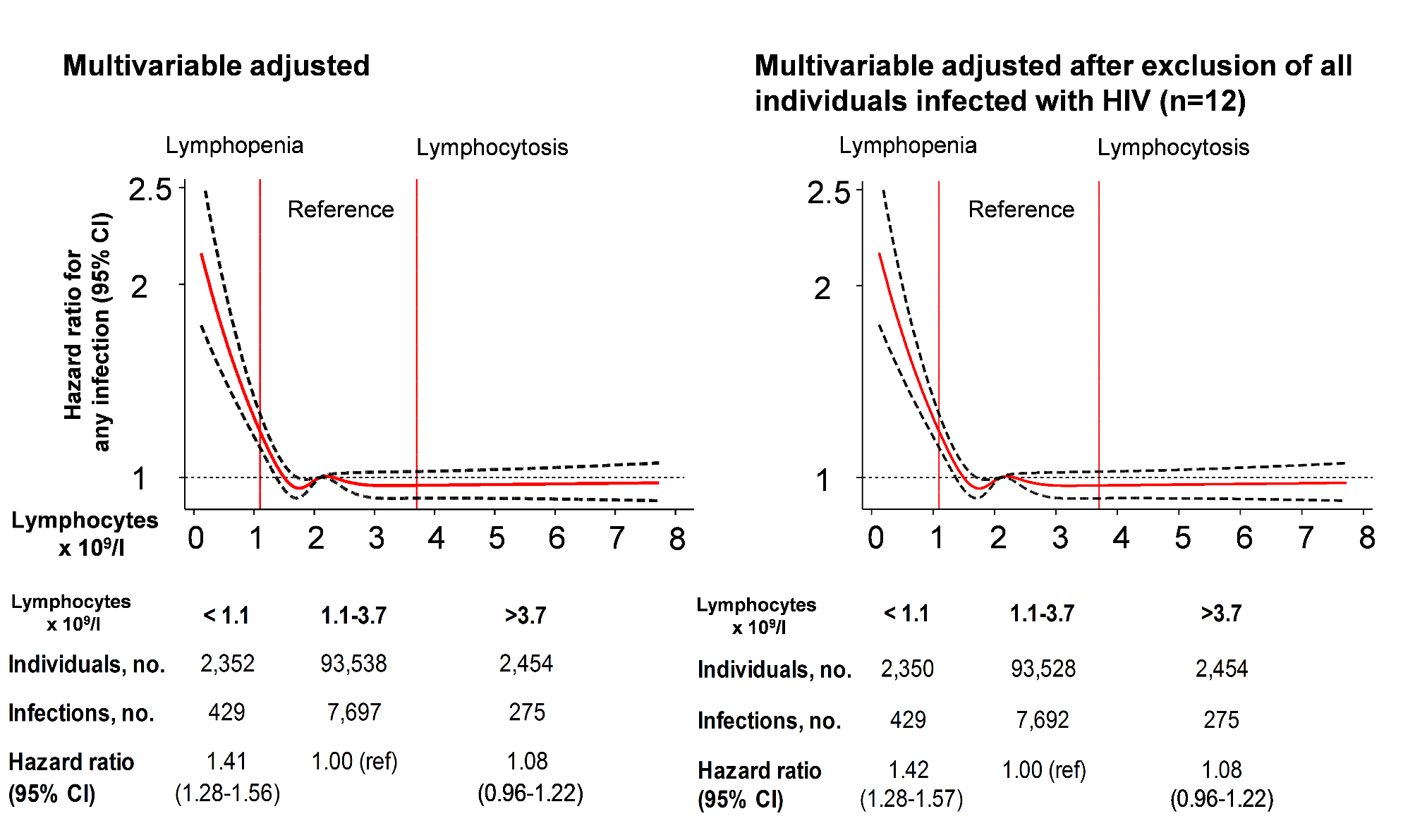


**Fig F:** **Risk of any infection as a function of lymphocyte count for individuals from the Copenhagen General Population Study, after exclusion of individuals infected with HIV/AIDS.** Multivariable adjusted risk of any infection for individuals from the Copenhagen General Population Study with lymphopenia (lymphocyte count <1.1 x 10^9^ /l) and lymphocytosis (>3.7 x 10^9^/l) compared to individuals with lymphocytes in the reference range (1.1-3.7 x 10^9^ /l). Multivariable adjustment includes all covariates listed in Table 1 except age, but with age as the underlying timescale. Results are presented in all individuals (left) and when excluding individuals infected with HIV/AIDS (n=12) defined as a diagnosis of HIV/AIDS either before date of examination or during follow-up (right). The median lymphocyte value of 2.1 x 10^9^/l was set as reference for the continuous model. When categorizing lymphocyte counts, lymphopenia was defined as a lymphocyte count below the 2.5^th^ percentile, the reference category was defined as a lymphocyte count between the 2.5^th^ and 97.5^th^ percentile, and lymphocytosis was defined as a lymphocyte count above the 97.5^th^ percentile.

**
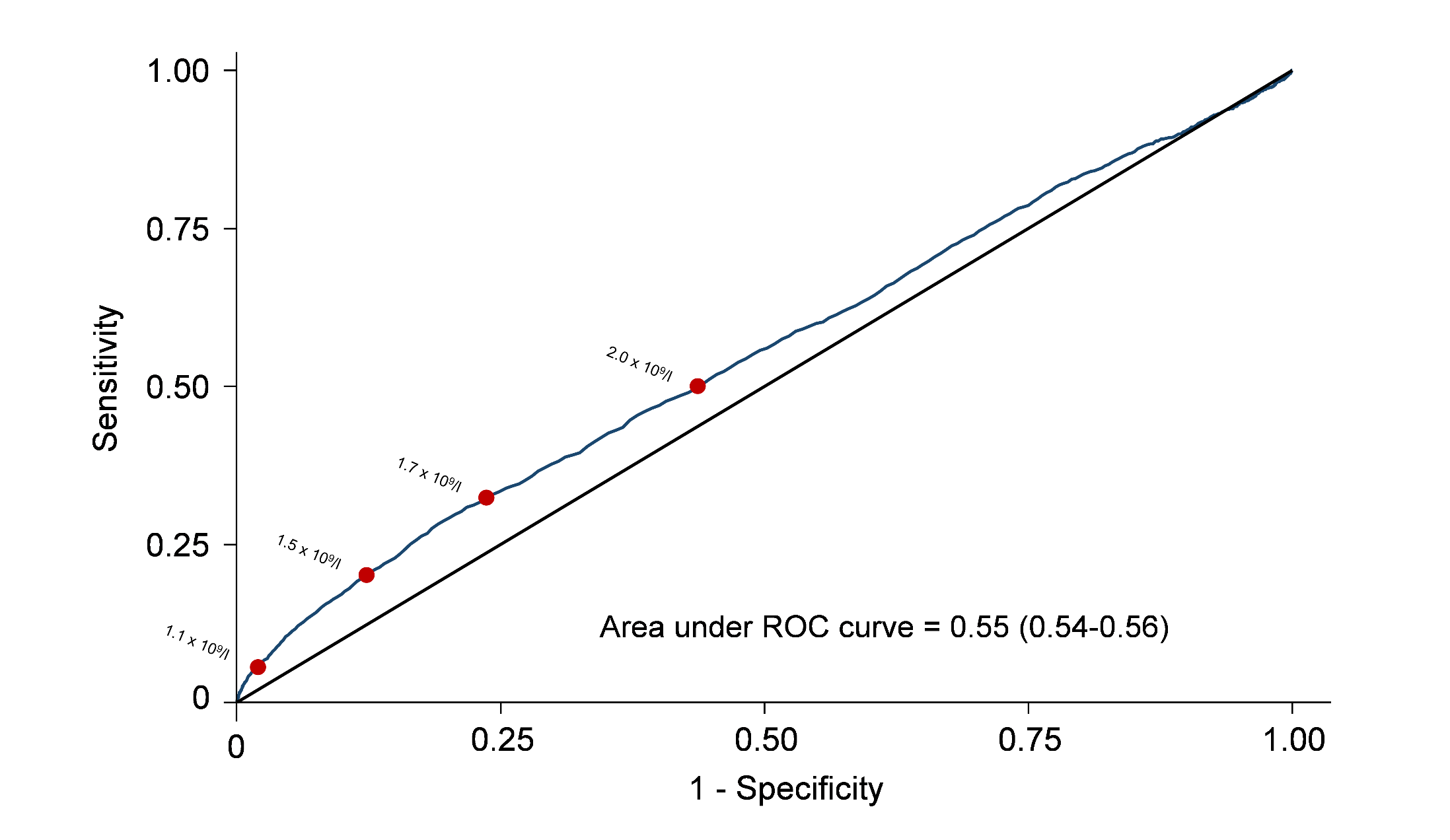
**

**Fig G: Receiver operating characteristic curve of sensitivity as a function of 1-specificity for cutoffs of lymphocyte counts to predict individual risk of future hospitalization due to an infection.** Infectious events in the first 2 years of follow-up are included. Individuals diagnosed with immunodeficiency, hematologic disease, and/or autoimmune disease were not included, since these individuals have a well-known high risk of infection.

**
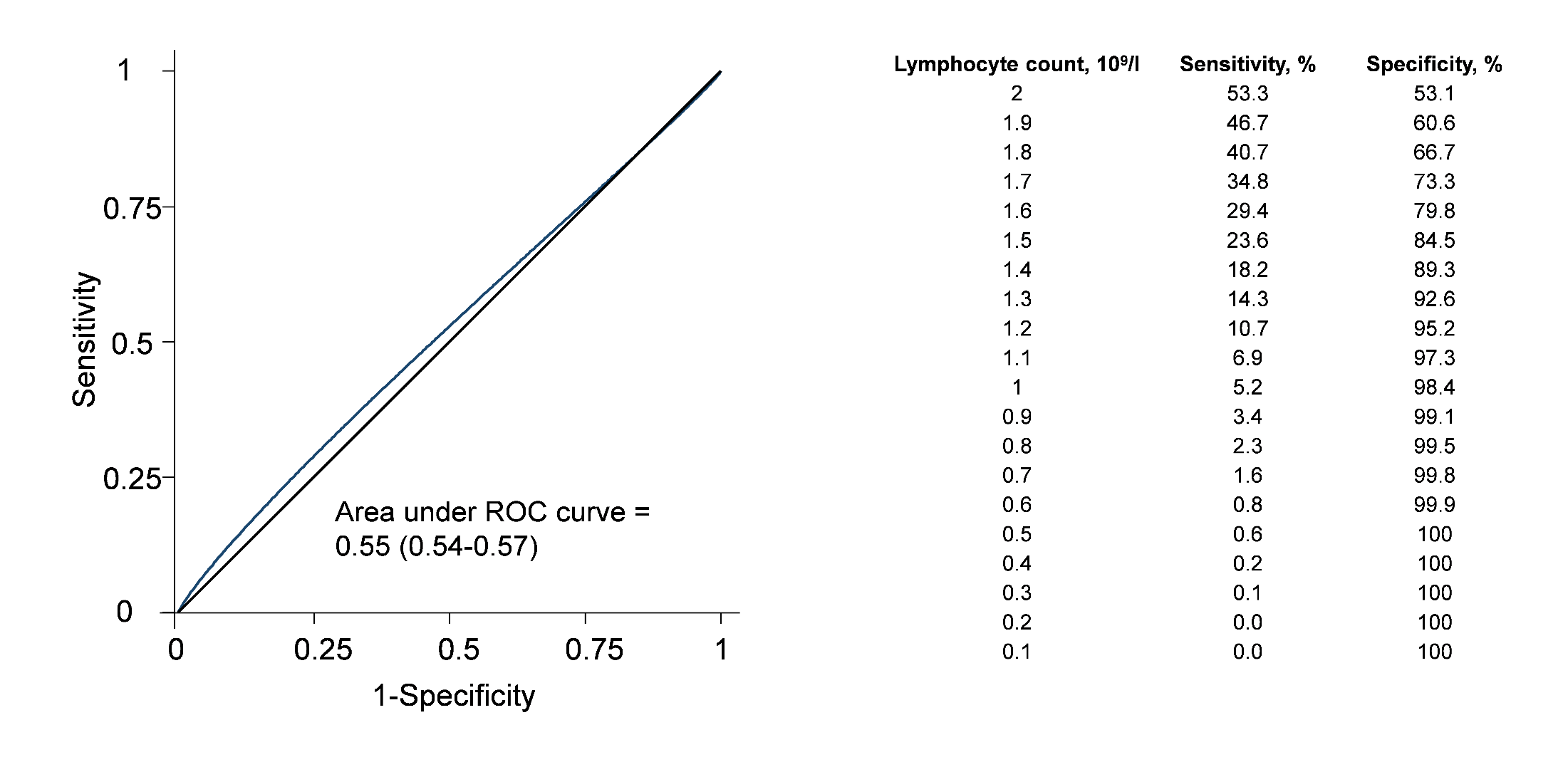
**

**Fig H:** **Receiver operating characteristic curve of sensitivity as a function of 1-specificity for cutoffs of lymphocyte counts, adjusted for age, sex and smoking status, to predict individual risk of future hospitalization due to an infection.** Infectious events in the first 2 years of follow-up are included. Individuals diagnosed with immunodeficiency, hematologic disease, and/or autoimmune disease were not included, since these individuals have a well-known high risk of infection. Smoking status was defined as either smokers or nonsmokers (former smokers and never smokers combined). The table show sensitivity and specificity for lymphocyte counts in the range 0.1-2.0 x 10^9^/l.

**References**

1. Eaton WW, Pedersen MG, Nielsen PR, Mortensen PB. Autoimmune diseases, bipolar disorder, and non-affective psychosis. Bipolar Disord. 2010;12: 638–646. doi:10.1111/j.1399-5618.2010.00853.x
