# Supplementary material for: Lymphopenia and risk of infection and infection-related death in 98,344 individuals from a prospective Danish population-based study
Source: PLoS Med. 2018 Nov 1;15(11):e1002685. doi: 10.1371/journal.pmed.1002685 (PMC6211632; doi:10.1371/journal.pmed.1002685)
Supplement: S1 STROBE Checklist — (DOCX) [file pmed.1002685.s002.docx]

**STROBE Statement**

|  | Item No | Recommendation | Check | Paragraph/Section |
| --- | --- | --- | --- | --- |
| **Title and abstract** | 1 | (*a*) Indicate the study’s design with a commonly used term in the title or the abstract | \| **√** \| \| --- \| | Title  Abstract, Methods and Findings, paragraph 1 |
|  |  | (*b*) Provide in the abstract an informative and  balanced summary of what was done and what was found | \| **√** \| \| --- \| | Abstract, Methods and Findings (paragraph 1 and 2) |
| Introduction | | |  |  |
| Background/rationale | 2 | Explain the scientific background and rationale  for the investigation being reported | \| **√** \| \| --- \| | Introduction (paragraph 1) |
| Objectives | 3 | State specific objectives, including  any prespecified hypotheses | \| **√** \| \| --- \| | Introduction (paragraph 3) |
| Methods | | |  |  |
| Study design | 4 | Present key elements of study design  early in the paper | \| **√** \| \| --- \| | Participants, in Methods |
| Setting | 5 | Describe the setting, locations, and relevant dates, including periods of recruitment, exposure, follow-up, and data collection | \| **√** \| \| --- \| | Participants, in Methods |
| Participants | 6 | (*a*) Give the eligibility criteria, and the sources and methods of selection of participants.  Describe methods of follow-up | \| **√** \| \| --- \| | Participants, in Methods |
|  |  | (*b*) For matched studies, give matching criteria and  number of exposed and unexposed | \|  \| \| --- \| | N/A |
| Variables | 7 | Clearly define all outcomes, exposures, predictors, potential confounders, and effect modifiers. Give diagnostic criteria, if applicable | \| **√** \| \| --- \| | Covariates (paragraph 1 and 2), Blood lymphocyte count (paragraph 2), Infectious disease endpoints (paragraph 3) and Comorbidities (paragraph 1 and 2) in Methods. |
| Data sources/ measurement | 8* | For each variable of interest, give sources of data and details of methods of assessment (measurement). Describe comparability of assessment methods if there is more than one group | \| **√** \| \| --- \| | Covariates (paragraph 1 and 2), Blood lymphocyte count (paragraph 1 and 2), Infectious disease endpoints (paragraph 1-3) and Comorbidities (paragraph 1 and 2) in Methods. |
| Bias | 9 | Describe any efforts to address potential sources of bias | \| **√** \| \| --- \| | Covariates (paragraph 1 and 2), and Comorbidities (paragraph 1 and 2) in Methods.  *.* |
| Study size | 10 | Explain how the study size was arrived at | \| **√** \| \| --- \| | Participants in Methods and Methods and Findings in Abstract |
| Quantitative variables | 11 | Explain how quantitative variables were handled in the analyses. If applicable, describe which groupings were chosen and why | \| **√** \| \| --- \| | Covariates in Methods. |
| Statistical methods | 12 | (*a*) Describe all statistical methods, including those used to control for confounding | \| **√** \| \| --- \| | Statistical analyses (paragraph 1-4) and Sensitivity analyses (paragraph 1-3) in Methods |
|  |  | (*b*) Describe any methods used to examine  subgroups and interactions | \| **√** \| \| --- \| | Sensitivity analyses (paragraph 1-3) in Methods |
|  |  | (*c*) Explain how missing data were addressed | \| **√** \| \| --- \| | Statistical analyses (paragraph 5) in Methods |
|  |  | (*d*) If applicable, explain how loss to follow-up  was addressed | \| **√** \| \| --- \| | Participants in Methods |
|  |  | (*e*) Describe any sensitivity analyses | \| **√** \| \| --- \| | Sensitivity analyses in Methods |
| Results | | |  |  |
| Participants | 13* | (a) Report numbers of individuals at each stage of study—eg numbers potentially eligible, examined for eligibility, confirmed eligible, included in the study, completing follow-up,  and analysed | \| **√** \| \| --- \| | Figure 1 |
|  |  | (b) Give reasons for non-participation at each stage | \|  \| \| --- \| | N/A |
|  |  | (c) Consider use of a flow diagram | \| **√** \| \| --- \| | Figure 1 |
| Descriptive data | 14* | (a) Give characteristics of study participants  (eg demographic, clinical, social) and information on exposures and potential confounders | \| **√** \| \| --- \| | Participants in Methods |
|  |  | (b) Indicate number of participants with missing data for each variable of interest | \| **√** \| \| --- \| | Statistical analyses (paragraph 5) in Methods |
|  |  | (c) Summarise follow-up time  (eg, average and total amount) | \| **√** \| \| --- \| | Lymphopenia and risk of infection (paragraph 1) in Results |
| Outcome data | 15* | Report numbers of outcome events or  summary measures over time | \| **√** \| \| --- \| | Lymphopenia and risk of infection (paragraph 1) in Results |
| Main results | 16 | (*a*) Give unadjusted estimates and, if applicable, confounder-adjusted estimates and their precision (eg, 95% confidence interval). Make clear which confounders were adjusted for and why they were included | \| **√** \| \| --- \| | Figures 3-9, Fig B-H in S1 Appendix, Figure Legends, Covariates Statistical analyses (paragraph 4) in Methods |
|  |  | (*b*) Report category boundaries when continuous variables were categorized | \| **√** \| \| --- \| | Figures 3-4 and 9, Fig B-D and F in S1 Appendix |
|  |  | (*c*) If relevant, consider translating estimates of relative risk into absolute risk for a meaningful time period | \|  \| \| --- \| | N/A |
| Other analyses | 17 | Report other analyses done—eg analyses of subgroups and interactions, and sensitivity analyses | \| **√** \| \| --- \| | Figures 5-7, Fig E-F in S1 Appendix, Stratified analyses and Sensitivity analyses in Results |
| Discussion | | |  |  |
| Key results | 18 | Summarise key results with reference to study objectives | \| **√** \| \| --- \| | Discussion (paragraph 1) |
| Limitations | 19 | Discuss limitations of the study, taking into account sources of potential bias or imprecision. Discuss both direction and magnitude of any potential bias | \| **√** \| \| --- \| | Strengths and limitations in Discussion |
| Interpretation | 20 | Give a cautious overall interpretation of results considering objectives, limitations, multiplicity of analyses, results from similar studies, and other relevant evidence | \| **√** \| \| --- \| | Comparison with other studies and Possible explanations and implications for clinicians in Discussion |
| Generalisability | 21 | Discuss the generalisability (external validity)  of the study results | \| **√** \| \| --- \| | Strengths and limitations in Discussion*.* |
| Other information | | |  |  |
| Funding | 22 | Give the source of funding and the role of the funders for the present study and, if applicable, for the original study on which the present article is based | \| **√** \| \| --- \| | *There was no specific funding for the present study.* |

*Give information separately for exposed and unexposed groups.
