# Supplementary material for: Lymphopenia and risk of infection and infection-related death in 98,344 individuals from a prospective Danish population-based study
Source: PLoS Med. 2018 Nov 1;15(11):e1002685. doi: 10.1371/journal.pmed.1002685 (PMC6211632; doi:10.1371/journal.pmed.1002685)
Supplement: S1 Text — (PDF) [file pmed.1002685.s003.pdf]

Undersøgernr.______
(do not fill out)

ØBUS P Nr.: ____________
(do not fill out)

HERLEV / ØSTERBROUNDERSØGELSEN

The Copenhagen General Population Study

Questionnaire

In this questionnaire we will ask you to answer some questions about your health and lifestyle. **We ask you to answer all questions.** The questions are answered by ticking the box that is most appropriate. Naturally, all answers will be treated with strict confidentiality.

Name			
Address			
Post code & city			
Telephone no.		Cpr-no.	

		Yes	No
1.	Do you experience pain or tightness in your chest when you are in a hurry, or when you use stairs?	☐	☐
2.	Have you ever been hospitalized due to a heart attack ?	☐	☐
3.	Have you ever had a heart attack without being admitted to the hospital ?	☐	☐
4.	Have you ever had heart by-pass surgery?	☐	☐
5.	Have you ever had a blockage removed from the blood vessels of your heart (angioplasty/PCI/PTCA) ?	☐	☐
6.	Have you ever had a thrombosis (blood clot) in your legs ?	☐	☐
7.	Have you ever had a thrombosis (blood clot) in your lungs ?	☐	☐
8.	Do you have shortness of breath, when you are in a hurry or go up a hill ?	☐	☐
9.	Do you have more shortness of breath, when walking at normal pace on a straight road compared to people your own age ?	☐	☐
10.	Do you sometimes have to stop and catch your breath, when walking down the street at your own pace ?	☐	☐
11.	Do you sometimes wake up at night due to shortness of breath or strenuous breathing ?	☐	☐
12.	Do you have shortness of breath when taking a bath or when getting dressed ?	☐	☐

		Yes	No
13.	Do you have shortness of breath when sitting quietly or resting ?	☐	☐
14.	Are you often troubled by shortness of breath ?	☐	☐
15.	Do you sometimes cough during physical activity ?	☐	☐
16.	Do you cough up mucus (in the mornings or during the day) as long as 3 consecutive months a year ?	☐	☐
17.	Have you been exposed to dust or fumes over long periods of time in your job ?	☐	☐
18.	Do you sometimes experience wheezing (high-pitched whistling sound during breathing) ?	☐	☐
	**If yes**: During a cold ?	☐	☐
	During physical activity ?	☐	☐
	Without any cause ?	☐	☐
19.	Do food, medicines, grass, flowers, animal hair or anything else give you ?		
	Asthma ?	☐	☐
	Hay fever ?	☐	☐
	Eczema ?	☐	☐
20.	Did you have asthma, hay fever, or eczema as a child ?	☐	☐
21.	Do you have asthma ?	☐	☐
	**If yes**: How many years have you had asthma? Years: ☐		

22. Have you had acute bronchitis or pneumonia within the last 10 years that lead to consultation of a doctor or absence from work?

No	Yes, 1-5 times	Yes, 6-10 times	Yes, more than 10 times
☐	☐	☐	☐

		Yes	No
23.	Have you within the last 10 years had:		
	a) paralysis, weakness, or coordination difficulties of your face, arms, or legs?	☐	☐
	b) blindness or loss of vision in one or both eyes ?	☐	☐
	c) abnormal speech, difficulty with retrieving or pronouncing words?	☐	☐

	Yes	No
24. Have you ever had a stroke or a haemorrhage in the brain ?	☐	☐
25. Do you experience pain in one or both legs:		
a) when you start walking ?	☐	☐
b) when you have walked for a while ?	☐	☐
**If yes**: Do you have to stop, when you have walked for a while ?	☐	☐
**If yes**: Does the pain stop, when you stop walking ?	☐	☐
26. Have you had an acute episode of fever, bronchitis, or bladder infection within the last 4 weeks ?	☐	☐
27. Do you have diabetes ?	☐	☐

If yes: How old were you, when you were diagnosed? ☐ years

28. Do you or have you had cancer ?	☐	☐

If yes: what type: ____________________________

How old were you at the time of diagnosis ? ☐ years

29. Do you or have you had other chronic diseases ?	☐	☐

If yes: what type: ______________________________

How old were you at the time of diagnosis ? ☐ years

30. Are you or have you ever been a blood donor ?	☐	☐

If yes: How many years have been a donor ? ☐ years

Only for women – Men should proceed to question 37

31. How old were you, when your menstruations began ? ☐ years

	Yes	No
32. Have your menstruations stopped ?	☐	☐

If yes: How old were you when your menstruations stopped ? ☐ years

33. How many abortions have you had ? ☐

34. How many children have you had? No.: [ ]

35 How old were you at your first delivery ? [ ] years

		Yes	No
36.	Have you breastfed ?	☐	☐

If yes: Number of months total: [ ] months

37.	Do you smoke ?	☐	☐
	**If no**: Have you previously smoked ?	☐	☐

If you have never smoked please proceed to question 44

38. How many years have you smoked ? [ ] years

39. How old were you when you began smoking ? Age: [ ] years

40. If you have stopped smoking, how old were you when you stopped ? Age: [ ] years

41. If you smoke or have smoked, how much is/was you average consumption of:

Cigarettes without filter	No. per day:	[ ]
Cigarettes with filter	No. per day:	[ ]
Cheroots	No. per day:	[ ]
Cigars	No. per day:	[ ]
Pipe tobacco	Packets of 40/50 g per week	[ ]

		Yes	No
42.	Do you or did you inhale ?	☐	☐
43.	Do you use a nicotine substitution (chewing gum, patch etc.) ?	☐	☐

If yes: How many years have you used it ? [ ] years

44. How many hours a day are you exposed to passive smoking [ ] hours

45. What is your average **consumption per week** of:

Whole milk : [ ] glasses Semi-skimmed milk [ ] glasses Skimmed milk [ ] glasses

Coffee: [ ] cups Tea: [ ] cups Cola: [ ] × ½ L

Cola light: [ ] × ½ L Soft drinks: [ ] ×½ L Diet soft drinks: [ ] × ½ L

46. How often do you drink:

	Never/ almost never	Several times a month	Several times a week	Daily/ almost daily	Average **per week**	
Beer	☐	☐	☐	☐	Bottles:	☐
White wine	☐	☐	☐	☐	Glasses:	☐
Red wine	☐	☐	☐	☐	Glasses:	☐
Dessert wines	☐	☐	☐	☐	Glasses:	☐
Spirits/liquors	☐	☐	☐	☐	Units:	☐

	Yes	No
47. If you drink beer, is it mainly with your meals ?	☐	☐
48. If you drink wine, is it mainly with your meals ?	☐	☐
49. If you drink spirits, is it mainly with your meals ?	☐	☐

50. How many meals do you eat per day ? No.: ☐

51. How many slices of bread do you eat per day ?
(One slice =1/2 slice ”rugbrød”, 1 slice of white bread, 1 ”knækbrød” or 1/2 a bun) No.: ☐

52. What type of fat du you usually put on your bread ? (**only one answer**)

Nothing	Butter	Kærgården	Plant marg.	Minarine	Other
☐	☐	☐	☐	☐	☐

53. On average, how many **times a week** do you eat the following spreads/fillings **on bread** ?

Meats: ☐ Liver pâté: ☐ Fish: ☐ Cheese: ☐

54. On average, how many **times a week** do you eat the following types of main courses ?

Beef/veal: ☐ Pork: ☐ Poultry: ☐

Fish: ☐ Fastfood: ☐

55. What types of fats do you **usually** use for preparing main courses ? (**only one answer**)

Nothing	Butter	Kærgården	Marg.	Plant marg.	Minarine	Oil	Andet
☐	☐	☐	☐	☐	☐	☐	☐

56. How often do you eat vegetables as a snack, as a part of breakfast or lunch, or as a main ingredient in main courses ? (**only one answer**)

Almost never	1-3 × a month	1-2 × a week	3-4 × a week	5-6 × a week	1 × a day	2-3 × a day	>3 × a day
☐	☐	☐	☐	☐	☐	☐	☐

57. How often do you eat fruit (a whole piece of fruit or part of a fruit) ? (**only one answer**)

Almost never	1-3 × a month	1-2 × a week	3-4 × a week	5-6 × a week	1 × a day	2-3 × a day	>3 × a day
☐	☐	☐	☐	☐	☐	☐	☐

58. How long is you education ? ☐ years
(including from primary school to university level)

59. What type of education have you completed after leaving school ? (**only one answer**)

- ☐ None
- ☐ Under education
- ☐ A short education (up to 3 years with books)
- ☐ Vocational training or similar (1-3 years)
- ☐ Higher eduaction (≥3 years, e.g. teacher, nurse or similar)
- ☐ University

60. What type of employment have you had the longest after finishing your education ? (**only one answer**)

- ☐ Self-employed
- ☐ Skilled worker
- ☐ Unskilled worker
- ☐ Salaried employee / white-collar worker
- ☐ Housewife / working at home
- ☐ No employment (unemployed or pensioner)

61. Do you live:

- ☐ With spouse/companion
- ☐ Alone
- ☐ With others

62. How many children do you have ? ☐

63. How many persons in the household including yourself ? No.: ☐

64. Do you have a:

	Yes	No
Dog	☐	☐
Cat	☐	☐
Other pets	☐	☐

If yes: What pets: ________________________________

65. Are you:

- ☐ Married/in a relationship
- ☐ Not married
- ☐ Separated/divorced
- ☐ Widow/widower

66. What was the **total income** of your **household** before tax last year ? (**only one answer**)

- ☐ Less than 100.000 kr.
- ☐ Between 100.000 kr. and 200.000 kr.
- ☐ Between 200.000 kr. and 400.000 kr.
- ☐ Between 400.000 kr. and 600.000 kr.
- ☐ Between 600.000 kr. and 800.000 kr.
- ☐ More than 800.000 kr.

67. How many people in your household have contributed to the income ? ☐

	Yes	No
68. Do you often feel nervous or stressed ?	☐	☐
69. Do you often feel tired ?	☐	☐
70. Have you had the feeling that you are not accomplishing much lately ?	☐	☐
71. Has it become more difficult for you to complete tasks that require complete focus/concentration lately?	☐	☐
72. Do you have a feeling of hopelessness ?	☐	☐
73. Do you feel in good health ?	☐	☐

74. Indicate your PHYSICAL ACTIVITY DURING WORK **within the last year** (should also be answered by housewives, students, unemployed, while pensioners should proceed to question 75). (**only one answer**)

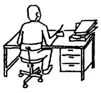

I. Primarily sitting most of the time
e.g. desk job, housewife without children and with a maid ☐

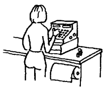

II. Sitting and standing, sometimes walking
e.g. shop assistent, teacher, housewife who does all washing and cleaning herself without small children ☐

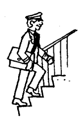

III. Mostly walking, sometimes lifting
e.g. mailman, healthcare worker, housewife who does all washing and cleaning herself with 1 or more small children ☐

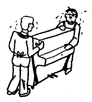

IV. Heavy labour
e.g. movers, construction workers ☐

	Yes	No
**If you ticked III or IV**: Do you often lift heavy loads ?	☐	☐

75. Indicate your LEISURE TIME PHYSICAL ACTIVITY (including transport to and from work) **within the last year** (**only one answer**)

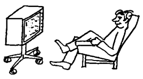

I. Almost completely physically inactive or light physical activity up to 2 hours a week.
e.g. reading, television, cinema ☐

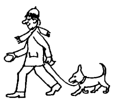

II. Light physical activity from 2-4 hours a week.
e.g. walks, biking, light gardening, light exercise ☐

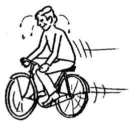

III. Light physical activity for more than 4 hours a week or more intense physical activity from 2-4 hours a week
e.g. fast walking and/or fast cycling, laborious gardening, heavy exercise with sweating or breathlessness ☐

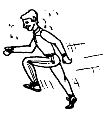

IV. Intense physical activity for more than 4 hours a week or regular intense training potentially with participation in competitions several times a week ☐

	Yes	No
**If you ticked III or IV**: Does you traning involve weight-lifting or heavier strength/weight training ?	☐	☐
76. Have you markedly changed your exercise habits within the last year ?	☐	☐

If Yes: To more exercise ☐ To less exercise ☐

77. How many **biological** siblings do you have ? No.: ☐

78.

Have your **biological** parents or **biological** siblings had:	**Mother**			**Father**			**One or more siblings**		
	Yes	No	Unknown	Yes	No	Unknown	Yes	No	Unknown
A heart attack ?	☐	☐	☐	☐	☐	☐	☐	☐	☐
A stroke / brain haemorrhage ?	☐	☐	☐	☐	☐	☐	☐	☐	☐
Asthma ?	☐	☐	☐	☐	☐	☐	☐	☐	☐
Diabetes ?	☐	☐	☐	☐	☐	☐	☐	☐	☐
High blood pressure ?	☐	☐	☐	☐	☐	☐	☐	☐	☐
High cholesterol ?	☐	☐	☐	☐	☐	☐	☐	☐	☐
Cancer ?	☐	☐	☐	☐	☐	☐	☐	☐	☐
What type of cancer	-----------------------			-----------------------			-----------------------		

If your parent or siblings have had a heart attack or a stroke/brain haemorrhage how old were they the first time ?

Mother: ☐ years

Father: ☐ years

Siblings: ☐ years

79.

What education did your (biological) parents have ?	Mother	Father
None or a short education	☐	☐
Vocational training or similar (1-3 years)	☐	☐
Higher eduaction (≥3 years, e.g. teacher, nurse and so forth)	☐	☐
University	☐	☐

80. Within the last 12 months, have you been to:

	Yes	No	If yes:	
A general practitioner (GP) (Praktiserende læge) ?	☐	☐	No. times:	☐
A specialist ?	☐	☐	No. times:	☐
An emergency room or an out patient clinic?	☐	☐	No. times:	☐
Been submitted to a hospital ?	☐	☐	No. days:	☐
