# Supplementary material for: Lymphopenia and risk of infection and infection-related death in 98,344 individuals from a prospective Danish population-based study
Source: PLoS Med. 2018 Nov 1;15(11):e1002685. doi: 10.1371/journal.pmed.1002685 (PMC6211632; doi:10.1371/journal.pmed.1002685)
Supplement: S2 Text — (PDF) [file pmed.1002685.s004.pdf]

Name:___________________________ ØBUS P Nr: ____________

(do not fill out)

Do you daily or almost daily ingest

	Yes	No	How Long		Drug name
Hjertemagnyl/ Magnyl/ Codymagnyl/Acetylsalicylsyre	☐	☐	☐	years	____________
Bloodpressure pills	☐	☐	☐	years	____________
" (more)	☐	☐	☐	years	____________
" (more)	☐	☐	☐	years	____________
Heart medication	☐	☐	☐	years	____________
" (more)	☐	☐	☐	years	____________
" (more)	☐	☐	☐	years	____________
Diuretics (water tablets)	☐	☐	☐	years	____________
Medication against high cholesterol	☐	☐	☐	years	____________
Pills for arthritis	☐	☐	☐	years	____________
Sleeping pills	☐	☐	☐	years	____________
Sedatives or pills for depression	☐	☐	☐	years	____________
Pills for acidity or acid reflux	☐	☐	☐	years	____________
Medication for asthma/bronchitis Spray/inhaler)	☐	☐	☐	years	____________
Insulin	☐	☐	☐	years	____________
Other medication for diabetes	☐	☐	☐	years	____________
Contraceptive pills	☐	☐	☐	years	____________
Hormon supplemnts for menopause	☐	☐	☐	years	____________
Pills/drops for eye disease	☐	☐	☐	years	____________
Painkillers or pain relieving medicine	☐	☐	☐	years	____________
Weight-loss/anti-obesity pills	☐	☐	☐	years	____________
Other medication	☐	☐	☐	years	____________
Vitamine pills	☐	☐	☐	years	____________
Herbal medicine/Dietary supplements	☐	☐	☐	years	____________
Herbal medicine/Dietary supplements	☐	☐	☐	years	____________
Herbal medicine/Dietary supplements	☐	☐	☐	years	____________

V
